# From energy metabolic homeostasis to immune remodeling: the role and therapeutic potential of STING signaling in the tumor microenvironment

**DOI:** 10.1186/s12964-026-02974-1

**Published:** 2026-06-12

**Authors:** Kui Zhao, Xiaohua Wen, Lanyu Zheng, Chaoran Wang, Ziyu Kuang, Siyuan Cui, Na Wang, Jinli Zhu, Fanming Kong

**Affiliations:** 1 Department of Oncology, First Teaching Hospital of Tianjin University of Traditional Chinese Medicine, Tianjin University of Traditional Chinese Medicine, Tianjin, 300381 China; 2 National Clinical Research Center for Chinese Medicine, Tianjin, 300381 China; 3 Tianjin Cancer Institute of Traditional Chinese Medicine, Tianjin, 300381 China; 4 Tianjin University of Traditional Chinese Medicine, Tianjin, 301617 China

**Keywords:** STING signaling, Tumor microenvironment, Energy metabolism, Immune remodeling, Anti-tumor therapy

## Abstract

The stimulator of interferon genes (STING) pathway is a core regulatory axis of innate immunity. By sensing cytoplasmic DNA, it activates the transcription of type I interferons and pro-inflammatory cytokines, playing a pivotal role in mediating anti-tumor immune responses. Accumulating evidence indicates that the biological functions of STING signaling extend well beyond immune regulation, engaging in extensive and dynamic crosstalk with energy metabolic homeostasis within the tumor microenvironment (TME). The bidirectional interplay forms a central mechanism that shapes the overall immune landscape of the TME. Here, we systematically review the mechanisms by which STING signaling interacts with four core energy metabolic pathways—glycolysis, oxidative phosphorylation, lipid metabolism, and glutamine metabolism—as well as key metabolic regulators. We clarify the critical role of STING as a hub that integrates cellular metabolic stress, energy status, and innate immune responses. Building on these findings, we further elaborate on the pathological significance of crosstalk between STING signaling and the energy metabolic network in TME remodeling. Finally, we highlight combinatorial anti-tumor therapeutic strategies that target key metabolic-immune intersections within this regulatory network, offering a theoretical framework and practical insights for developing novel therapies. In conclusion, the intricate and dynamic crosstalk between STING signaling and the core energy metabolic network establishes it as a critical node linking innate immunity with metabolic homeostasis, and strategically targeting these metabolic-immune intersections represents a promising avenue for developing more effective, combination-based anti-tumor therapies.

## Introduction

Cancer biology is gradually shifting from a cancer cell-centric paradigm to a more comprehensive framework that situates cancer cells within a stromal cell network comprising fibroblasts, vascular cells, and immune cells involved in inflammatory processes, together forming the tumor microenvironment (TME) [1]. During early tumorigenesis, tumor-suppressive immune cells within the TME effectively recognize and eliminate malignant cells. However, as the disease progresses, cancer cells acquire diverse strategies to evade immune attack and actively remodel the TME into an immunosuppressive state—a critical step in cancer progression [2].

Within this dynamic process, metabolic reprogramming serves not only as an adaptive strategy for cancer cells to survive under stressors such as hypoxia and nutrient deprivation, but also as a core mechanism by which they actively orchestrate immune responses [3, 4]. Since the discovery of the Warburg effect, which revealed that cancer cells preferentially utilize aerobic glycolysis for energy production, our understanding of tumor energy metabolism has substantially broadened, now encompassing the global remodeling of glucose, lipid, and amino acid metabolic networks [5]. Notably, although glycolysis represents a hallmark metabolic feature of cancer cells, many retain functional mitochondrial respiration to support their survival and proliferation [6].

This metabolic reprogramming disrupts energy homeostasis among immune cells within the TME, thereby enabling immune evasion [7]. Studies have shown that cancer cells systematically outcompete immune cells for energy substrates such as glucose, limiting T-cell proliferation and metabolism, and consequently promoting tumor progression [7–9]. Moreover, this process confers a metabolic advantage to immunosuppressive populations, including regulatory T cells (Tregs) and M2-type macrophages, further suppressing anti-tumor immunity. Thus, metabolic reprogramming represents not only a core adaptive strategy of cancer cells but also a pivotal mechanism sustaining immunosuppression [10, 11]. In light of this, restoring energy metabolic homeostasis in the TME has emerged as a promising therapeutic approach for cancer treatment.

In recent years, the cyclic GMP-AMP synthase-stimulator of interferon genes (cGAS-STING) signaling pathway has garnered significant attention as a central hub linking intracellular stress responses with innate immunity. The pathway detects nuclear and mitochondrial DNA (mtDNA) released due to genomic instability or mitochondrial stress, thereby triggering the production of type I interferons (IFN-I) and inflammatory cytokines that modulate the tumor immune microenvironment [12]. Notably, emerging evidence indicates that the cGAS-STING pathway, beyond its canonical role in mediating immune activation, directly influences cellular energy metabolism, positioning it as a key node in the crosstalk between immunity and metabolism [13, 14]. These insights offer a new perspective on the mechanisms underlying the disruption and remodeling of energy homeostasis within the TME.

In this review, we systematically delineate the bidirectional regulatory network linking the STING signaling pathway and energy metabolic homeostasis within the TME. We summarize the reciprocal regulatory mechanisms between STING signaling and core metabolic pathways, including glycolysis, oxidative phosphorylation (OXPHOS), lipid metabolism, and glutaminolysis, and analyze how the interplay between key energy homeostasis regulators and the cGAS-STING axis influences immune cell remodeling. Finally, we discuss current challenges in the field, including remodeling tumor immunometabolism, optimizing combination therapeutic strategies, and identifying predictive biomarkers, to offer new insights for the development of innovative anti-tumor therapies targeting the immunometabolic axis.

### Overview of the cGAS-STING pathway

The cGAS-STING signaling pathway plays a pivotal role in cancer immunotherapy by orchestrating the expression of IFN-I and the secretion of chemokines in tumor cells, thereby promoting the establishment of an immune-activated TME [15, 16]. Structurally, the cGAS protein comprises an unstructured N-terminal domain and a C-terminal catalytic domain [17]. It recognizes exogenous or endogenous double-stranded DNA (dsDNA) that enters the cytoplasm, such as DNA released upon viral infection, genomic instability, or DNA damage induced by radiotherapy and chemotherapy [18, 19]. The C-terminal nucleotidyltransferase domain constitutes the catalytic core and contains three functional modules: a dsDNA recognition domain, a catalytic core, and a zinc-binding motif [20]. Bridged by zinc ions, dsDNA binds to two cGAS molecules, facilitating the formation of a cGAS-dsDNA complex with 2:2 stoichiometry and thereby activating cGAS enzymatic activity [21]. In addition, the intrinsically disordered N-terminal domain contributes to the spatiotemporal precision of cGAS signaling through phase separation [22, 23]. Upon activation, cGAS undergoes a conformational change and catalyzes the synthesis of the second messenger 2′,3′-cyclic GMP-AMP (2′,3′-cGAMP) from ATP and GTP [23].

**Fig. 1:**
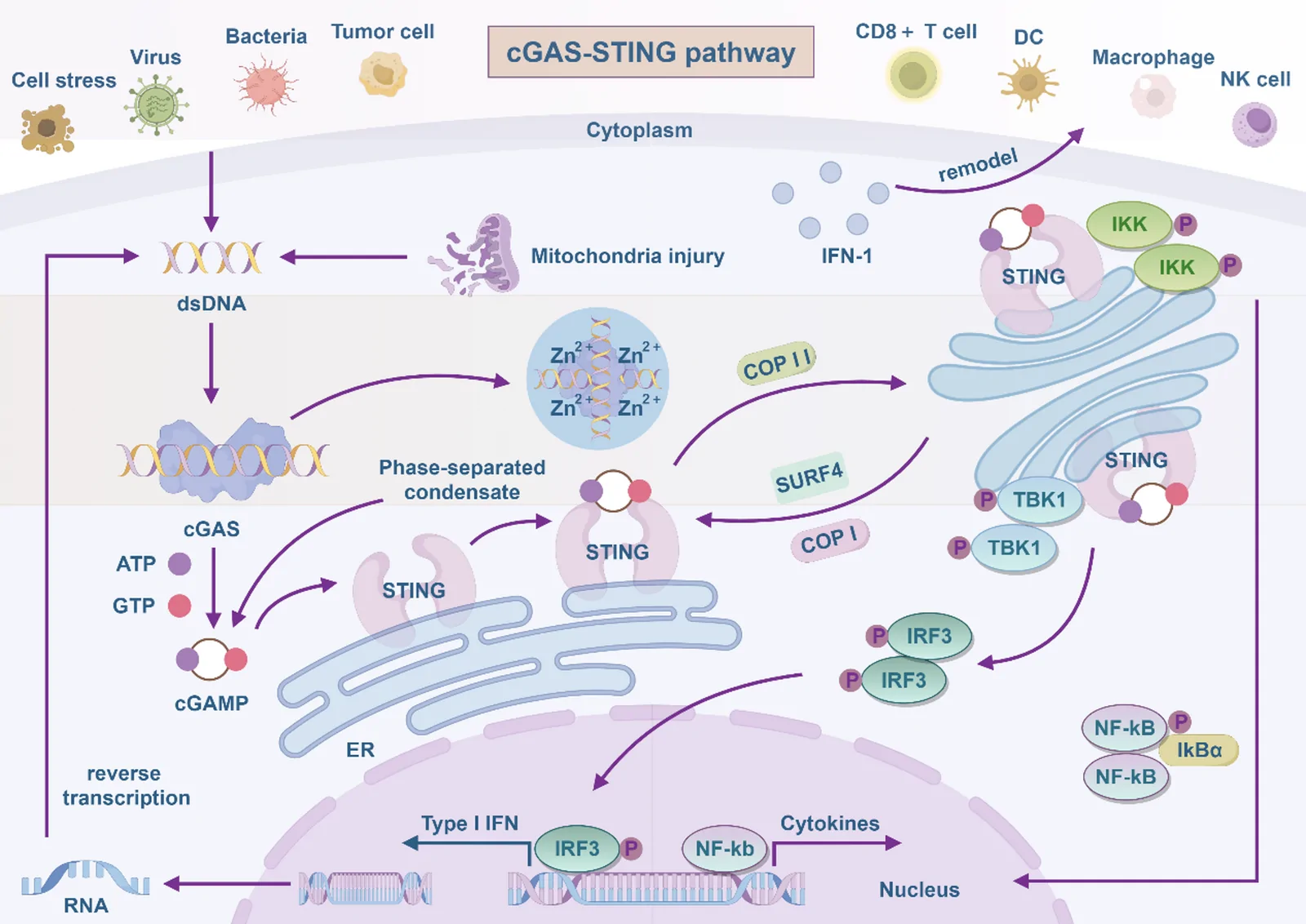
Overview of the canonical cGAS-STING signaling pathway. cGAMP: Cyclic GMP-AMP; cGAS: Cyclic GMP-AMP synthase; COPI/COPII: Coat protein complex I/II; DC: Dendritic cell; dsDNA: Double-stranded DNA; ER: Endoplasmic reticulum; IFN-I: Type I interferon; IKK: IκB kinase; IRF3: Interferon regulatory factor 3; NF-κB: Nuclear factor kappa-B; NK: Natural killer; STING: Stimulator of interferon genes; SURF4: Surfeit locus protein 4; TBK1: TANK-binding kinase 1

Upon binding to 2′,3′-cGAMP, STING, an endoplasmic reticulum (ER)-resident protein, undergoes high-order oligomerization into a tetramer, which is subsequently transported from the ER to the Golgi apparatus via a trafficking mechanism mediated by the coat protein complex II (COPII) coat complex and ADP-ribosylation factor (ARF) GTPases [24, 25]. In parallel, COPI-coated vesicles, with the assistance of the adaptor protein surfeit locus protein 4 (SURF4), can recycle a subset of STING from the Golgi apparatus back to the ER [26, 27]. At the Golgi apparatus, STING is palmitoylated at two cysteine residues (Cys88 and Cys91), an event that recruits TANK-binding kinase 1 (TBK1) and subsequently interferon regulatory factor 3 (IRF3). Specifically, TBK1 phosphorylates IRF3, promoting its dimerization. Dimerized IRF3 then translocates to the nucleus, where it initiates the transcription of IFN-I and interferon-stimulated genes (ISGs) (Fig. 1) [28]. IFN-I has been shown to promote dendritic cells (DCs) maturation and migration, enhance antigen presentation, activate tumor-associated macrophages (TAMs) and natural killer (NK) cells, and induce the expression of chemokines such as C-X-C motif chemokine ligand (CXCL) 9 and CXCL10, thereby recruiting and activating CD8⁺ cytotoxic T lymphocytes (CTLs) [29, 30]. In parallel, the STING-TBK1 signaling axis activates the IκB kinase (IKK) complex, leading to the phosphorylation, ubiquitination, and degradation of IκBα. Liberating nuclear factor kappa-B (NF-κB) from sequestration, this cascade enables its nuclear translocation and subsequent transcription of target genes [29]. However, in both solid tumors and hematologic malignancies, cGAS-STING plays a double-edged role, particularly when the pathway is abnormally and persistently activated [30–32]. For example, the core-binding factor subunit beta and smooth muscle myosin heavy-chain fusion (CBFβ-SMMHC) protein induces cytoskeletal co-option of mitochondrial constriction (CCMC) through cytoskeletal remodeling, generating mitochondrial-derived vesicles, which subsequently activate the cGAS-STING pathway and drive leukemogenesis [31]. On the other hand, activation of the STING pathway synergizes with chemotherapy and immunotherapy, triggering an anti-tumor immune response characterized by elevated levels of IFN-Ⅰ and pro-inflammatory cytokines [30].

Notably, the traditional view of the cGAS-STING pathway as a mere “DNA sensor” is insufficient to account for its multifaceted roles within the TME. Accumulating evidence suggests that this pathway may also function as an energy metabolism sensor, engaging directly or indirectly with key metabolic pathways and regulatory molecules to form a dynamic energy-regulatory network. Beyond monitoring the metabolic and energy status of cells, the network actively shapes the fate of both immune and tumor cells. Elucidating this emerging role of STING in energy homeostasis will offer novel therapeutic strategies and intervention targets for remodeling the TME.

### STING signaling and mitochondrial quality control

Mitochondria serve as the central hub of cellular energy metabolism and play a pivotal role in orchestrating dynamic interactions among diverse immune cell populations within the TME [33–35]. Accumulating evidence indicates that aberrant mitochondrial protein assembly, impaired dynamics, and dysregulated mitophagy contribute to mitochondrial dysfunction, which in turn promotes the release of mtDNA into the cytoplasm, thereby triggering or amplifying cGAS-STING pathway activation [36–38]. Mitophagy, a critical quality control mechanism for eliminating damaged mitochondria, is essential for maintaining mitochondrial protein homeostasis and dynamic balance, thus effectively restraining cGAS-STING activation. Competent mitophagy, particularly PTEN-induced putative kinase 1 (PINK1)-mediated mitophagy, facilitates the timely clearance of damaged mitochondria and limits mtDNA leakage, thereby exerting negative feedback on excessive cGAS-STING signaling and preserving immune homeostasis [39, 40]. Notably, the cGAS-STING pathway itself effectively induces mitophagy. Upon activation, STING upregulates dynamin-related protein 1 (DRP1) to promote mitochondrial fission while concurrently downregulating the fusion proteins mitofusins 1/2 (MFN1/2), thereby initiating mitophagy [41, 42].

In a STING-independent manner, cGAS directly binds to the core autophagy regulator Beclin1, displacing its inhibitor Rubicon, which accelerates autophagosome formation and facilitates the clearance of cytoplasmic DNA [43, 44]. Furthermore, TBK1, a core kinase downstream of cGAS-STING, directly phosphorylates ras-related protein 7 A (RAB7A) to initiate ubiquitin-dependent mitophagy via the PINK1-Parkin pathway. TBK1 also phosphorylates multiple autophagy receptors, including optineurin (OPTN) and nuclear domain 10 protein 52 (NDP52), enhancing their affinity for both ubiquitin chains and microtubule-associated protein 1 light chain 3 (LC3), thereby amplifying mitochondrial clearance signals [45, 46]. Concurrently, mitophagy mediates metabolic recycling, a process through which amino acids, fatty acids, and nucleotides derived from autophagic degradation are reintroduced into the metabolic pool to sustain energy homeostasis, enabling cells to remain metabolically replete and survive under nutrient stress [47]. Notably, this recycling process indirectly restricts non-specific STING activation induced by energy depletion, representing an additional layer of autophagy-STING crosstalk [48].

Collectively, the bidirectional feedback loop between mitochondrial quality control and STING signaling is critical for preventing the establishment of a chronic inflammatory TME. From a translational perspective, precise modulation of the intensity and temporal dynamics of mitophagy, specifically by targeting the PINK1/Parkin axis or TBK1-mediated phosphorylation of autophagy receptors in combination with STING agonists, holds promise for reversing metabolic stress-induced immune evasion and enhancing the responsiveness of solid tumors to immunotherapy [49].

### STING signaling and key energy metabolic pathways

Dysregulation of energy metabolism within the TME drives tumor progression and remodels intercellular communication. This metabolic imbalance arises from competitive reprogramming of core pathways, including glycolysis, OXPHOS, lipid metabolism, and glutaminolysis, by tumor cells, which deplete the microenvironment of essential nutrients such as glucose and glutamine, thereby limiting their availability for infiltrating immune cells. Such reprogramming sustains tumor proliferation while establishing a metabolic niche that favors immune evasion. Emerging evidence indicates that STING signaling, beyond its canonical immune role, is intimately intertwined with these metabolic remodeling processes through reciprocal regulatory mechanisms. In this section, we delineate the dynamic interactive network between STING signaling and core metabolic pathways, and explore how this network governs cell fate, shapes the TME, and influences disease progression.

### Glycolysis

A bidirectional, mutually reinforcing feedback loop operates between STING signaling and glycolysis. Initially driven by the energetic support that glycolysis provides for STING pathway activation, ATP generated through glycolysis supplies the essential energy substrates required for STING-TBK1-IRF3 signal transduction [50, 51]. Conversely, activated STING signaling upregulates glycolytic rate-limiting enzymes such as 6-phosphofructo-2-kinase/fructose-2,6-bisphosphatase 3 (PFKFB3) by inducing key transcription factors, including Hypoxia-inducible factor 1-α **(**HIF-1α) and Signal transducer and activator of transcription 3 (STAT3), thereby driving glycolytic metabolic reprogramming [50, 52, 53]. Thus, a self-reinforcing cycle is established: STING activation promotes glycolysis, and enhanced glycolysis in turn fuels more robust STING signaling.

Mitochondrial function and integrity serve as a critical hub governing the direction and outcome of this crosstalk. Accumulating evidence indicates that multiple factors inducing mitochondrial dysfunction, such as pyruvate carboxylase deficiency, carnitine acetyltransferase knockdown, or intracellular NAD⁺ depletion, trigger the release of mtDNA into the cytoplasm, accompanied by a metabolic shift toward glycolysis [54–58]. This phenomenon may be attributed to activated STING signaling stabilizing HIF-1α through increased mitochondrial reactive oxygen species (ROS), thereby further promoting glycolysis [59]. Thus, mitochondrial damage triggers inflammation and metabolic reprogramming via the STING pathway, and the resulting immune stress in turn exacerbates mitochondrial damage, collectively driving disease progression.

The crosstalk between STING signaling and glycolysis differentially shapes the TME depending on cell type. In the context of immune activation, the STING-glycolysis positive feedback loop promotes the antigen-presenting function of DCs and drives macrophage polarization toward the M1 phenotype, thereby enhancing anti-tumor immunity [50, 51]. Fructose-1,6-bisphosphatase 1 (FBP1), highly expressed in tumor cells, inversely activates the cGAS-STING-NF-κB pathway by inhibiting glycolysis, promoting secretion of immune factors such as IL-33, and further enhancing DC function [60]. Additionally, STING promotes glycolytic flux in DCs by activating hexokinase 2 (HK2) and pyruvate kinase M2 (PKM2) [50]. In stark contrast, a diametrically opposed regulatory mechanism operates in tumor cells. STING protein directly binds to and inhibits HK2 activity, a key glycolytic rate-limiting enzyme, thereby restricting aerobic glycolysis in tumor cells and promoting anti-tumor immunity in vivo [61]. This cell type-specific regulation of energy metabolism by STING signaling constitutes a core mechanism underlying its anti-tumor effects. Furthermore, since STING signaling regulates glycolysis, potentially affecting the expression of glyceraldehyde-3-phosphate dehydrogenase (GAPDH), GAPDH should not be used as a normalization tool when investigating the STING pathway under metabolic conditions within the TME [62].

Nevertheless, this crosstalk may also facilitate the formation of an immunosuppressive microenvironment and promote tumor immune evasion. In small cell lung cancer (SCLC), cancer-associated fibroblasts (CAFs) activate STING by promoting glycolysis in tumor cells, which increases the production of T cell chemokines. Concurrently, this interaction promotes colocalization of antigen-presenting CAFs (apCAFs) with CD8⁺ T cells, thereby sequestering CD8⁺ T cells and facilitating Tregs differentiation [63]. In the melanoma microenvironment, activation of STING signaling inhibits IFN-α production and CXCL10 chemokine expression in plasmacytoid dendritic cells (pDCs). In this context, tumor-derived immunosuppressive factors and metabolic shifts collectively drive pDC-mediated immune evasion [64]. Studies have also found that excessive activation of this signaling impairs the capacity of splenic DCs to activate CD8⁺ T cells, further suppressing anti-tumor immune responses [65].

### Oxidative phosphorylation

Dysfunction of mitochondrial OXPHOS and the consequent disruption of energy homeostasis are key upstream events driving STING signaling activation. Diverse stimuli that impair OXPHOS or induce mitochondrial dysfunction trigger the release of mtDNA into the cytoplasm, thereby activating the cGAS-STING pathway and eliciting extensive metabolic and immune remodeling [66]. For example, in senescent cells, dysfunctional mitochondria associated with the downregulation of nuclear-encoded mitochondrial OXPHOS genes drive the formation of cytoplasmic chromatin fragments via the ROS-JNK retrograde signaling pathway, thereby activating the cGAS-STING axis. This process may lead to inflammation and inhibit oncogene activation [67, 68]. In contrast, during normal mitosis, cGAS remains inactive when bound to nuclear chromatin, further illustrating that the impact of cGAS-STING signaling on transcriptional and immune programs is highly dependent on the subcellular localization of chromatin binding [69].

As the core genetic component of the OXPHOS system, mtDNA integrity is critical for maintaining mitochondrial function. Studies have shown that exposure to the chemical CEES impairs electron transport chain (ETC) function, leading to ROS accumulation and decreased mitochondrial membrane potential. Concomitantly, it downregulates mitochondrial transcription factor A (TFAM), exacerbates mtDNA damage, alters expression of mtDNA-encoded OXPHOS subunits, and activates the cGAS-STING-IRF3 pathway and subsequent inflammatory responses [57, 70]. Deficiencies in autophagy-related 16-like 1 (ATG16L1), downregulation of Carnitine acetyltransferase (CRAT) expression, and other perturbations similarly induce mitochondrial damage and mtDNA leakage—events essential for STING signaling activation and for maintaining the energy metabolic balance between OXPHOS and glycolysis [71, 72]. Notably, the cGAS-STING axis also exerts non-canonical functions in metabolic remodeling. For example, upon activation by mtDNA, cGAS independently stabilizes high expression of its interacting protein NDUFA4L2 in a STING-independent manner, thereby sustaining glycolytic reprogramming in tumor cells while inhibiting OXPHOS and the concomitant ROS production. This mechanism ultimately promotes tumor cell survival under stress conditions [73].

**Fig. 2:**
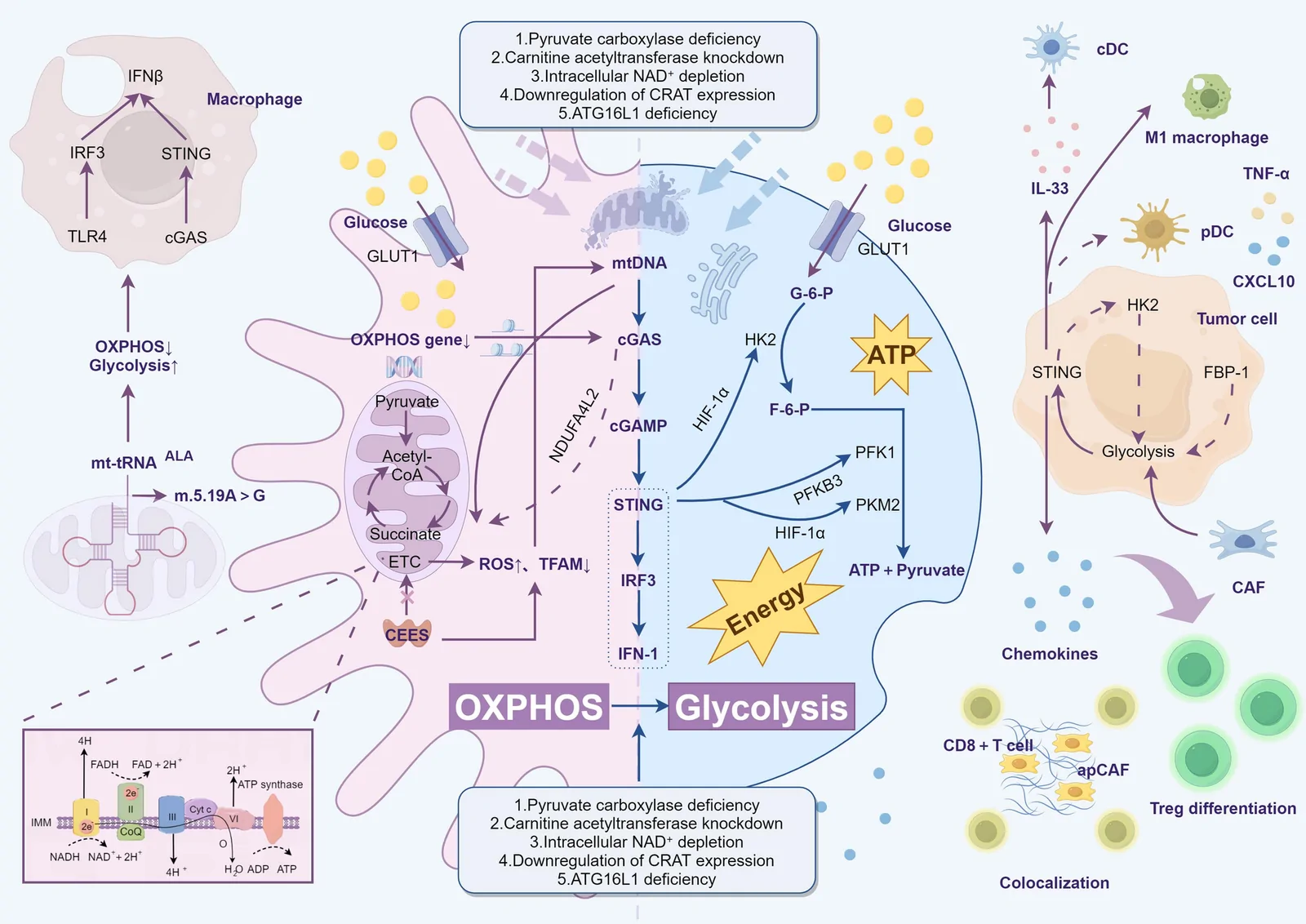
STING signaling regulates metabolic balance between glycolysis and oxidative phosphorylation. apCAF: Antigen-presenting CAF; ATG16L1: Autophagy-related 16-like 1; CAF: Cancer-associated fibroblast; cGAMP: Cyclic GMP-AMP; cDC: Conventional dendritic cell; CRAT: Carnitine acetyltransferase; CXCL10: C-X-C motif chemokine ligand 10; ETC: Electron transport chain; FAD: Flavin adenine dinucleotide; FBP-1: Fructose-1,6-bisphosphatase 1; FADH: Flavin adenine dinucleotide hydrate; G-6-P: Glucose-6-phosphate; GLUT1: Glucose transporter type 1; HIF-1α: Hypoxia-inducible factor 1-alpha; HK2: Hexokinase 2; IFN-I: Type I interferon; IL-33: Interleukin 33; IMM: Inner mitochondrial membrane; IRF3: Interferon regulatory factor 3; mtDNA: Mitochondrial DNA; mt-tRNA: Mitochondrial RNA; NAD⁺: Nicotinamide adenine dinucleotide (Oxidized); NADH: Nicotinamide adenine dinucleotide (Hydride); OXPHOS: Oxidative phosphorylation; PFK1: Phosphofructokinase-1; PFKFB3: 6-Phosphofructo-2-kinase/fructose-2,6-bisphosphatase 3; PKM2: Pyruvate kinase M2; ROS: Reactive oxygen species; STING: Stimulator of interferon genes; TFAM: Mitochondrial transcription factor A; TLR4: Toll-like receptor 4; TNF-α: Tumor necrosis factor-alpha

Metabolic intervention modulates cell fate by targeting this regulatory network. Arginine starvation induces α-ketoglutarate (α-KG) depletion and epigenetic silencing of metabolic genes, inhibiting OXPHOS and triggering tumor cell death. The consequent DNA damage and chromatin leakage activate cGAS-STING signaling, potentially enhancing anti-tumor efficacy [74]. Specific genetic defects likewise remodel immunometabolic responses. In macrophages harboring heteroplasmic mitochondrial tRNA mutations, reduced OXPHOS efficiency and disorganized cristae, accompanied by increased glycolysis, elicit a biphasic IFN-I response: an early Toll-like receptor 4 (TLR4)-IRF3 driven phase followed by sustained cGAS-STING activation from mitochondrial-derived nucleic acids, thereby exacerbating inflammatory pathology [75]. Fig. 2 illustrates the role of STING signaling in balancing glycolysis and OXPHOS.

### Lipid metabolism

STING directly binds and regulates key enzymes involved in lipid metabolism, extending beyond its canonical immune functions. In esophageal squamous cell carcinoma cells, STING binds to carnitine palmitoyltransferase 1 A (CPT1A), the rate-limiting enzyme of fatty acid oxidation (FAO), and disrupts its interaction with the deubiquitinase ubiquitin-specific protease 15 (USP15), thereby promoting CPT1A ubiquitination and degradation. The resulting FAO inhibition contributes to tumor suppression [76]. This regulatory mechanism is evolutionarily conserved: Drosophila STING interacts with acetyl-CoA carboxylase (ACC) and fatty acid synthase (FASN) to form a multi-enzyme complex that coordinately modulates lipid synthesis [77]. More importantly, STING serves as a central hub for systemic lipid metabolic homeostasis. The cholesterol sensor SREBP cleavage-activating protein (SCAP) recruits STING and TBK1 to the Golgi apparatus, activating downstream NF-κB signaling and ultimately driving lipolysis and hepatic lipid synthesis [78]. Loss of STING function abrogates this regulation, and STING-knockout mice exhibit sustained weight gain and elevated circulating triglycerides and cholesterol, confirming its critical role in systemic lipid metabolism [79].

Conversely, lipid metabolism dysregulation activates the cGAS-STING pathway. In Tregs, inhibition of the lipid chaperone FABP5 disrupts mitochondrial cristae structure, triggering mtDNA release and subsequent STING activation. Unexpectedly, this induces suppressive cytokines such as IL-10, enhancing Treg immunosuppressive function [80]. In triple-negative breast cancer, CPT1A inhibition causes lipid accumulation and mitochondrial damage, activating STING but promoting infiltration of anti-tumor neutrophils [81]. Peroxisome proliferator-activated receptor α (PPARα), a core lipid metabolism regulator, inhibits mitochondrial oxidative stress, thereby suppressing STING-NF-κB signaling and neutrophil extracellular traps (NETs) release, restricting tumor growth under high-fat diet conditions [82]. Thus, while lipid metabolic dysregulation triggers STING activation, downstream signaling direction depends on cell type, metabolic context, and microenvironmental factors. Identifying the molecular switches governing this directionality remains a critical question.

Lipid metabolism status and associated molecules actively modulate STING signaling. Liver X receptor (LXR) agonists inhibit STING by inducing the nuclease SMPDL3A to degrade cGAMP [83]. The lipid droplet-associated protein ancient ubiquitous protein 1 (AUP1) forms a complex with ubiquitin-conjugating enzyme E2 G2 (UBE2G2) to anchor STING to the ER, maintaining its resting state. Conversely, AUP1 loss reduces lipid accumulation, alters lipid metabolic gene expression, and triggers spontaneous STING activation [84]. The cholesterol metabolite 25-hydroxycholesterol suppresses STING activity [85]. Changes in lipid metabolic enzyme activity also modulate STING signaling: inhibition of fatty acid synthase or certain lipases attenuates STING, whereas sterol O-acyltransferase 1 (SOAT1) inhibition exerts species-specific effects, enhancing STING-dependent interferon induction in mouse cells but impairing it in human cells [86]. Notably, lipid metabolism and STING signaling are also coupled to mitochondrial function. The RNA-binding protein RBM43, induced by inflammatory signals, impairs mitochondrial biogenesis by inhibiting peroxisome proliferator-activated receptor γ coactivator 1-α (PGC1α) translation, whereas RBM43 knockout in adipocytes improves mitochondrial function and suppresses STING activation [87].

**Fig. 3:**
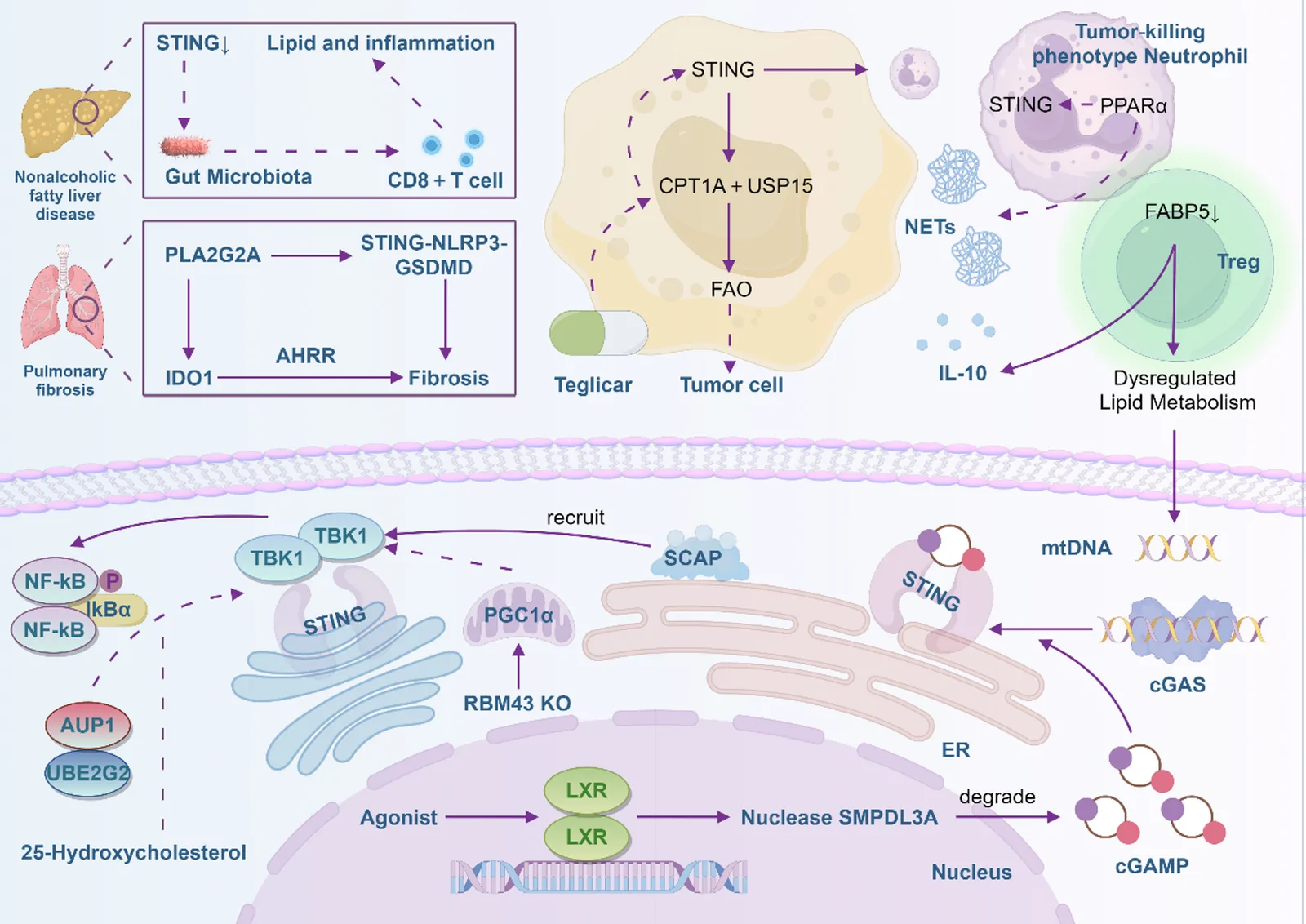
Crosstalk between STING signaling and lipid metabolism. AHRR: Aryl hydrocarbon receptor repressor; AUP1: Ancient ubiquitous protein 1; cGAMP: Cyclic GMP-AMP; cGAS: Cyclic GMP-AMP synthase; CPT1A: Carnitine palmitoyltransferase 1 A; ER: Endoplasmic reticulum; FAO: Fatty acid β-oxidation; LXR: Liver X receptor; mtDNA: Mitochondrial DNA; NETs: Neutrophil extracellular traps; NF-κB: Nuclear factor kappa-B; NLRP3: NOD-like receptor family pyrin domain containing 3; PGC1α: Peroxisome proliferator-activated receptor γ coactivator 1-α; PLA2G2A: Phospholipase A2 group IIA; PPARα: Peroxisome proliferator-activated receptor α; SCAP: SREBP cleavage-activating protein; STING: Stimulator of interferon genes; TBK1: TANK-binding kinase 1; Treg: Regulatory T cell; UBE2G2: Ubiquitin-conjugating enzyme E2 G2; USP15: Ubiquitin-specific protease 15

In metabolic and fibrotic diseases, the STING-lipid metabolism axis demonstrates further complexity. In non-alcoholic fatty liver disease (NAFLD), systemic STING deficiency alleviates hepatic lipid accumulation and inflammation, a mechanism partially attributed to altered gut microbiota composition and inhibition of CD8⁺ T cell activation, suggesting that chronic STING hyperactivation disrupts metabolic homeostasis [88]. In pulmonary fibrosis, secretory phospholipase A2 group IIA (PLA2G2A) induces pyroptosis and mitochondrial damage, activating the STING-NOD-like receptor family pyrin domain containing 3 (NLRP3)-Gasdermin D (GSDMD) axis and driving fibrotic progression. PLA2G2A may further exacerbate fibrosis by directly modulating lipid metabolism or activating indoleamine 2,3-dioxygenase 1 (IDO1) via aryl hydrocarbon receptor repressor (AHRR)-mediated inhibition of the aryl hydrocarbon receptor [89]. Collectively, these findings extend the STING-lipid metabolism regulatory network from tumor immunity to broader metabolic and fibrotic diseases, highlighting its pathological significance (Fig. 3).

### Glutamine metabolism

**Fig. 4:**
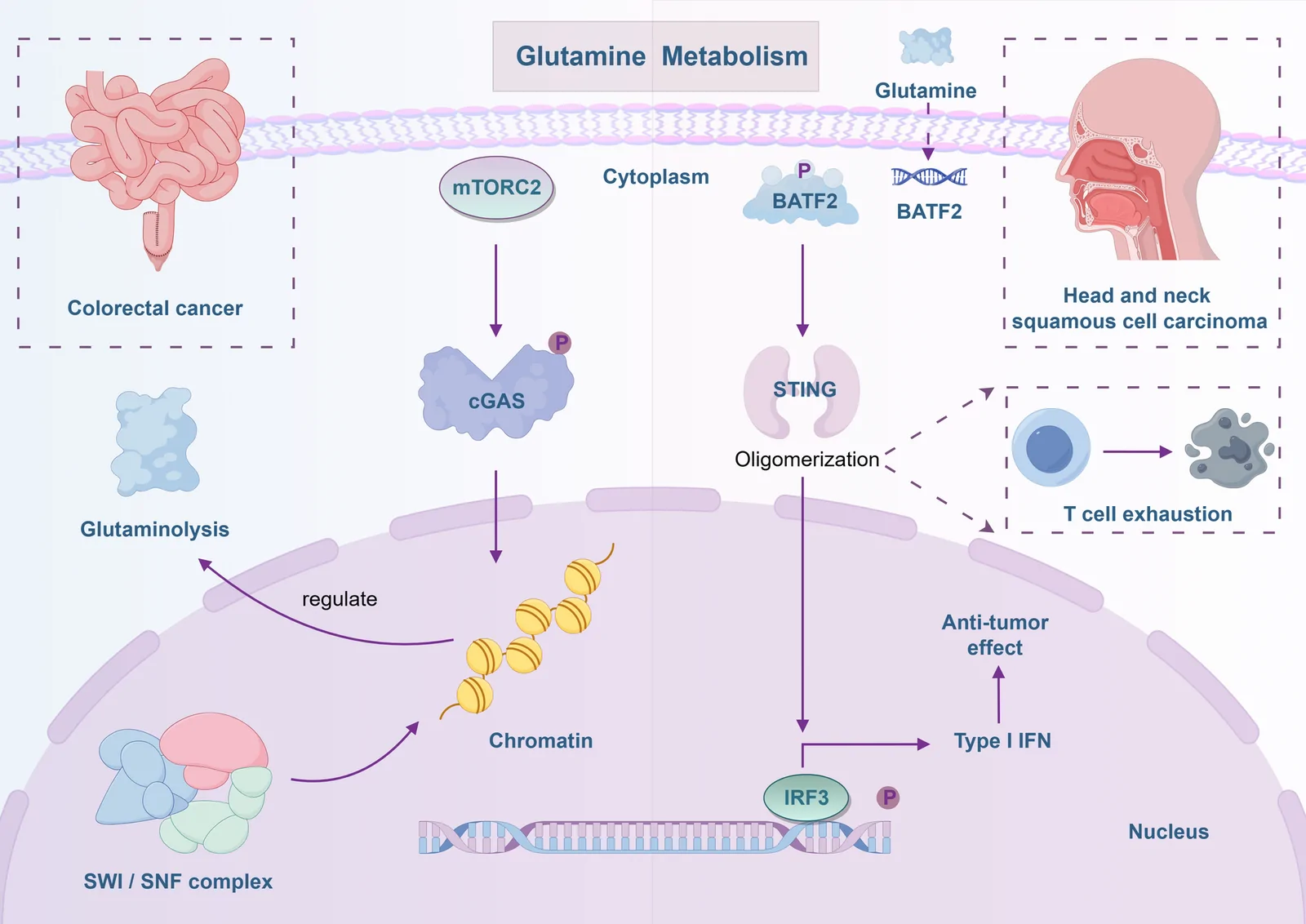
Crosstalk between STING signaling and glutamine metabolism. BATF2: Basic leucine zipper ATF-like transcription factor 2; cGAS: Cyclic GMP-AMP synthase; IRF3: Interferon regulatory factor 3; mTORC2: Mammalian target of rapamycin complex 2; STING: Stimulator of interferon genes

Recent studies have identified a STING-independent, non-canonical function of cGAS in regulating glutamine metabolism, revealing a previously unrecognized mechanism underlying tumor metabolic plasticity and therapeutic resistance. In colorectal cancer, mammalian target of rapamycin complex (mTORC) 2-mediated phosphorylation of cGAS at serine 37 drives its translocation to chromatin, where it recruits the SWI/SNF remodeling complex to specific genomic loci. This recruitment regulates genes involved in glutaminolysis and DNA replication, thereby directly influencing tumor energy homeostasis and therapeutic response [90]. In head and neck squamous cell carcinoma, the tumor suppressor basic leucine zipper ATF-like transcription factor 2 (BATF2) bridges glutamine metabolism and STING signaling. Phosphorylation of BATF2 at serine 227 promotes STING oligomerization, enhancing T cell effector function, alleviating exhaustion, and driving IFN-I-dependent anti-tumor immunity. However, high glutamine concentrations in the TME epigenetically silence BATF2 expression, impairing STING activation and restricting effector cell expansion, thereby compromising the efficacy of STING agonist-based therapies (Fig. 4) [91]. Collectively, these findings establish that cGAS and BATF2 couple glutamine metabolism with tumor immunity and chemotherapeutic response through non-canonical epigenetic regulation and STING-mediated signaling, offering new molecular targets and rational combination strategies to overcome therapeutic resistance.

### STING and key energy metabolic regulators

Maintaining energy homeostasis within the TME relies on the precise coordination of core energy-sensing and regulatory molecules. These include AMP-activated protein kinase (AMPK), which monitors energy status; mTOR, which integrates nutrient and growth signals; HIF-1α, which responds to hypoxic conditions; the oncogenes myelocytomatosis (MYC) and rat sarcoma (RAS), which drive anabolic metabolism; and the tumor suppressors p53 and liver kinase B1 (LKB1), which uphold metabolic homeostasis. Collectively, these components form a central regulatory network governing tumor metabolic reprogramming.

The crosstalk between the cGAS-STING pathway and this metabolic network extends far beyond the canonical role of STING as a “DNA sensor.” STING is activated by energy stress, oncogenic stress, or specific metabolic cues. In turn, it directly regulates key metabolic hubs such as mTOR and reciprocally modulates the expression or activity of factors including MYC, p53, and HIF-1α through transcriptional and epigenetic mechanisms. This multifaceted interplay positions STING as a central hub that integrates metabolic stress, energy status, and innate immune responses, thereby simultaneously shaping metabolic reprogramming and immune cell fate within the TME.

### AMPK

AMPK serves as a central guardian of cellular energy homeostasis. As a critical energy sensor, AMPK is activated by an elevated AMP/ATP or ADP/ATP ratio, coordinating diverse cellular processes including lipogenesis, glycolysis, the tricarboxylic acid (TCA) cycle, and mitochondrial dynamics [92, 93]. Its core function lies in switching on catabolic metabolism, such as glycolysis and fatty acid oxidation, to generate ATP under energy stress, while simultaneously switching off anabolic processes to rapidly restore energy balance [94–96]. Within the TME, the crosstalk between AMPK and the cGAS-STING pathway coordinately regulates both energy homeostasis and immune equilibrium.

First, AMPK functions as a key upstream activator of STING signaling. During antiviral immunity, activated AMPK directly phosphorylates TBK1 at serine 511, promoting assembly of the STING or mitochondrial antiviral signaling protein (MAVS) complex and enhancing IFN-I responses [97]. This stimulatory effect may occur independently of Unc-51-like kinase 1 **(**ULK1), the canonical downstream effector of AMPK, suggesting the involvement of a noncanonical pathway [98]. Further studies have revealed that the AMPK-TBK1 axis tightly links cellular glucose metabolic status to nucleic acid sensing, which is essential for host defense [97]. In addition, metabolic stress modulates STING signaling by affecting mitochondrial function. For example, under hyperglycemic conditions, activation of the Sirtuin 1 (SIRT1)-AMPK-PGC1α axis induces mitochondrial dysfunction, leading to mtDNA release via extracellular vesicles. These vesicles are taken up by adjacent fibroblasts, activating intracellular STING signaling and driving fibrosis and adverse tissue remodeling [99].

Second, AMPK often serves as a “brake” to suppress excessive STING activation and inflammatory responses [100]. The myokine Metrnl induces autophagy by activating the LKB1-AMPK-ULK1 pathway, allowing phosphorylated ULK1 to promote dephosphorylation and translocation of STING to mitochondria. Subsequent TNF receptor-associated factor 2 (TRAF2)-mediated ubiquitination and degradation inhibit the cGAS-STING pathway [101]. Similarly, fibroblast growth factor 21 (FGF21) prevents mtDNA leakage through AMPK-dependent mitophagy, suppressing cGAS-STING activation at its source [102]. Conversely, inhibition of AMPK activity releases this constraint, leading to excessive STING activation that promotes inflammation, fibrosis, or tumor immune escape [103, 104].

Furthermore, AMPK acts as a downstream effector of STING signaling, participating in functional feedback regulation. Upon cytoplasmic DNA detection by cGAS, activated STING triggers calcium release through transient receptor potential vanilloid 2 (TRPV2) channels, activating the calcium/calmodulin-dependent protein kinase kinase 2 (CAMKK2)-AMPK axis, which in turn helps maintain genome stability [105]. More importantly, studies suggest an intrinsic negative feedback loop between STING and the AMPK-ULK1 axis that prevents excessive and sustained innate immune signaling. Mechanistically, STING activation promotes dissociation of ULK1 from autophagy-related gene 1 (ATG1), and activated ULK1 subsequently phosphorylates STING and inhibits IRF3 to terminate signal transduction [106].

### mTOR

mTOR is a serine/threonine protein kinase that senses and integrates diverse inputs, including nutrients (amino acids, glucose), energy status (ATP/AMP ratio), growth factors, and stress signals, to precisely govern cell growth, proliferation, autophagy, and metabolic balance [107, 108]. As a central regulator of anabolic and catabolic programs, mTOR forms a dynamic, multi-layered regulatory network with the cGAS-STING pathway, shaping both cellular metabolism and immune phenotypes within the TME.

STING directly regulates mTOR signaling bidirectionally. On one hand, STING activation negatively modulates mTORC1 activity. Specifically, STING induces lipidation of gamma-aminobutyric acid type A receptor-associated protein (GABARAP) on vesicles. Modified GABARAP sequesters the folliculin (FLCN)-folliculin-interacting protein (FNIP) complex, blocking Ras-related GTP-binding (Rag) GTPase activation and subsequently inhibiting mTORC1. The resulting reduction in mTORC1 activity relieves repression of transcription factor EB (TFEB), driving lysosomal biogenesis [109, 110]. On the other hand, STING positively correlates with mTOR signaling under specific contexts. In T cells, STING promotes differentiation of T helper 1 (Th1) and T helper 9 (Th9) cells through parallel IRF3 and mTOR pathways, cooperatively enhancing anti-tumor immunity [111]. However, this positive regulation can be hijacked in disease states. In pancreatic ductal adenocarcinoma (PDAC), cGAS-STING activation in macrophages induces IFN-α production, elevates bone marrow stromal cell antigen 2 (BST2)-positive macrophages, and triggers CXCL7 secretion. CXCL7 subsequently activates the CXCR2 receptor and downstream RAC-alpha serine/threonine-protein kinase (AKT)-mTOR pathway in CD8⁺ T cells, driving T cell exhaustion and establishing an immunosuppressive microenvironment [112].

mTOR feeds back to regulate STING signaling, forming a core bidirectional interaction. Under stress conditions such as cellular senescence, elevated mTOR signaling represses TFEB, impairing lysosomal function. Consequently, damaged mitochondria and leaked mtDNA fail to be cleared, exacerbating persistent cGAS-STING activation and chronic inflammation [113]. In autoimmune diseases, including systemic lupus erythematosus (SLE), mTOR inhibition markedly reduces abnormally elevated STING and downstream molecule expression [114]. The E3 ubiquitin ligase membrane-associated RING-CH protein 1 (MARCH 1) weakens TBK1-mTOR interaction by promoting K63-linked ubiquitination of TBK1, thereby suppressing growth factor-mediated mTOR signaling. Notably, accelerated cell proliferation caused by MARCH 1 deficiency can be reversed by inhibitors of mTOR, STING, or TBK1 [115].

STING-mTOR crosstalk impacts tumorigenesis, therapeutic response, and immune microenvironment composition. In colorectal cancer cells (e.g., HCT116, SW480), STING promotes proliferation, invasion, and drug resistance via the AMPK-mTOR pathway [116]. In estrogen receptor-positive breast cancer, perinuclear-localized STING (pnSTING) correlates with favorable a prognosis and active immune infiltration, whereas low pnSTING expression associates with activated mTOR signaling and an immunosuppressive microenvironment [117]. In lung squamous cell carcinoma (LUSC), cytoplasmic DNA sensing is primarily mediated by DNA-dependent protein kinase (DNA-PK) rather than cGAS. DNA-PK enhances survival, migration, and chemoresistance through the leucine-zipper and sterile-α motif kinase (ZAK)-AKT-mTOR axis, likely by promoting glycolysis [118]. This finding reveals a DNA-sensing mechanism parallel to cGAS-STING that directly activates mTOR.

Additional metabolic and stress signals modulate both STING and mTOR, further expanding this regulatory network. Under high-glucose conditions, cGAS-STING activation by mitochondrial damage stimulates IRF3 through the extracellular signal-regulated kinase (ERK) 1/2-AKT-tuberous sclerosis complex-mTOR cascade [119]. Following DNA virus infection, the mTOR downstream effector ribosomal protein S6 kinase beta-1 (S6K1) acts as a scaffold via its kinase domain, forming a stable ternary complex with STING and TBK1. This assembly supports robust IRF3 activation, early antiviral gene expression, and subsequent adaptive immune responses, providing a framework for understanding how metabolic status fine-tunes antiviral defense [120].

### HIF-1

HIF-1 serves as a central hub enabling tumor cells to adapt to hypoxia and undergo metabolic reprogramming. Its active subunit HIF-1α drives the switch from OXPHOS to glycolysis by activating glycolysis-related genes, supporting tumor survival and therapeutic resistance under hypoxic conditions [121, 122]. HIF-1α engages in extensive bidirectional crosstalk with the cGAS-STING pathway, shaping immune cell function and modulating tumor progression.

Under basal conditions, HIF-1α constitutively binds the STING promoter and positively regulates its transcription [123]. Conversely, activated STING stabilizes HIF-1α through multiple mechanisms. In Brucella-infected macrophages, STING triggers endoplasmic reticulum stress and the unfolded protein response (UPR), during which inositol-requiring enzyme 1α (IRE1α) drives mitochondrial ROS production and stabilizes HIF-1α. Accumulated HIF-1α then redirects macrophage metabolism toward glycolysis and promotes M1-like polarization [124, 125]. Studies have also revealed that STING drives glycolysis by stabilizing HIF1α, and the resulting lactate directly induces histone lactylation at the HK2 gene loci, increasing chromatin accessibility and promoting IRF3 binding. This forms a positive feedback loop that amplifies glycolytic flux, thereby establishing a complete cascade from STING activation to metabolite production, epigenetic modification, and ultimately target gene regulation [126]. In PDAC cells, STING upregulates dual oxidase 2 (DUOX2) in an IRF3-dependent manner, elevating hydrogen peroxide (H₂O₂) production. H₂O₂ subsequently induces HIF-1α and vascular endothelial growth factor A (VEGF-A) under normoxic conditions and triggers DNA damage [127]. Similarly, in senescent retinal pigment epithelial cells, H₂O₂ upregulates VEGF via the STING-NF-κB-HIF-1α axis [128].

The co-activation of HIF-1α and STING governs metabolic reprogramming and functional polarization of immune cells, particularly in macrophages and DCs. In colorectal cancer-associated macrophages, nicotinamide phosphoribosyltransferase (NAMPT) deficiency destabilizes HIF-1α, reduces M2-like polarization, and paradoxically enhances STING signaling and IFN-I responses, exerting anti-tumor effects through cytotoxic T-cell activation [129]. STING agonists targeting triggering receptor expressed on myeloid cells 2 (TREM2) remodel macrophages toward a phagocytic M1-like phenotype by boosting the glycolysis-ROS-HIF-1α axis and inducing mitochondria-ER contacts [130]. In DCs, a positive feedback loop strengthens anti-tumor function: glycolytic metabolism facilitates STING activation, while activated STING further accelerates glycolysis through HIF-1α [50].

**Fig. 5:**
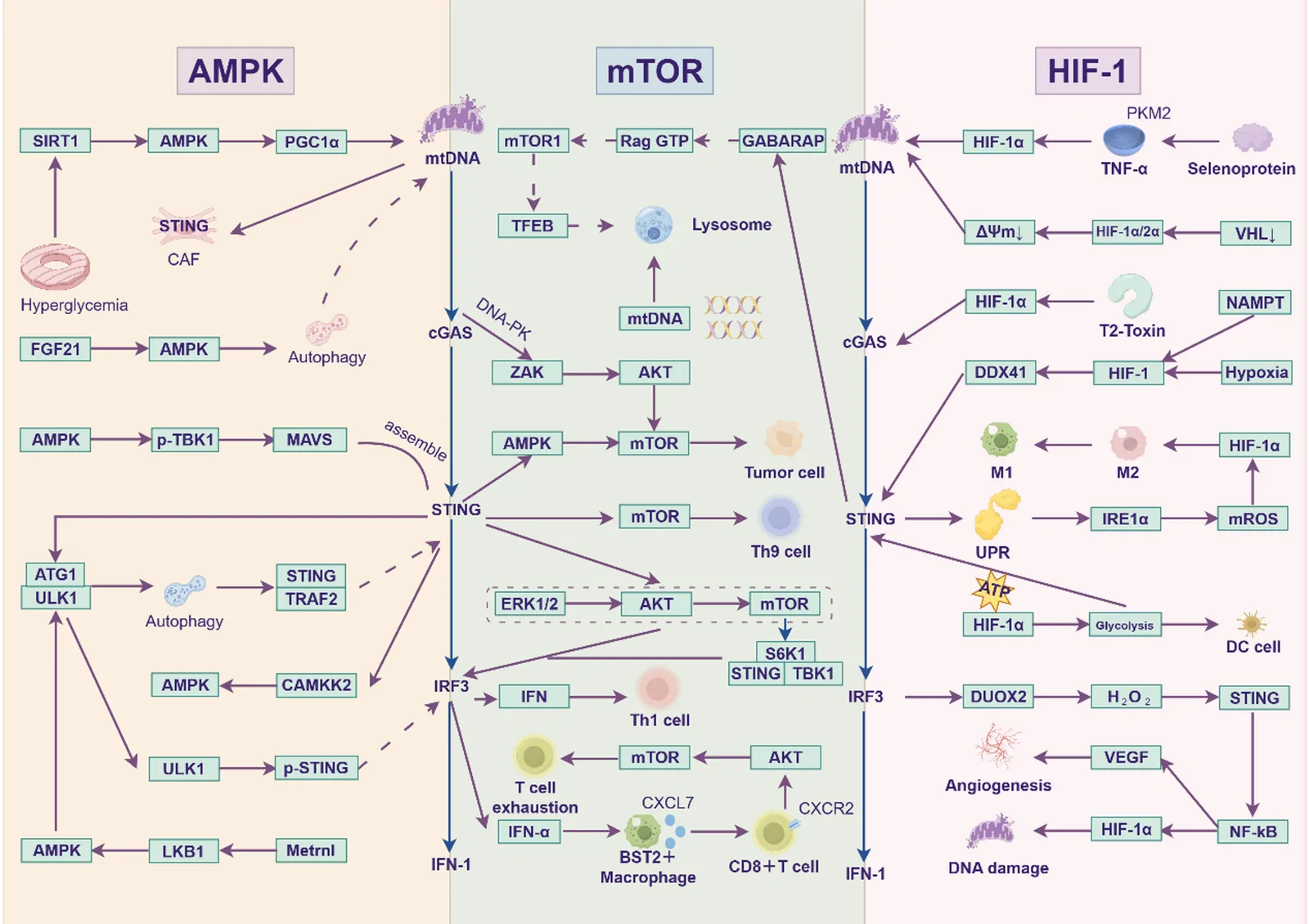
Crosstalk between STING signaling and key energy regulators, including AMPK, mTOR, and HIF-1. ATG 1: Autophagy-related gene 1; AKT: RAC-alpha serine/threonine-protein kinase; AMPK: AMP-activated protein kinase; CAMKK2: Calcium/calmodulin-dependent protein kinase kinase 2; CAF: Cancer-associated fibroblast; CXCL7: C-X-C motif chemokine ligand 7; DC: Dendritic cell; DDX41: DNA sensor DEAD-box helicase 41; DNA-PK: DNA-dependent protein kinase; DUOX2: Dual oxidase 2; ERK 1/2: Extracellular signal-regulated kinase 1/2; FGF21: Fibroblast growth factor 21; GABARAP: Gamma-aminobutyric acid type A receptor-associated protein; HIF-1α: Hypoxia-inducible factor 1-alpha; IFN-I: Type I interferon; IRE1α: Inositol-requiring enzyme 1α; IRF3: Interferon regulatory factor 3; LKB1: Liver kinase B1; MAVS: Mitochondrial antiviral signaling protein; mtDNA: Mitochondrial DNA; mTOR: Mammalian target of rapamycin; NAMPT: Nicotinamide phosphoribosyltransferase; NF-κB: Nuclear factor kappa-B; PGC1α: Peroxisome proliferator-activated receptor γ coactivator 1-α; PKM2: Pyruvate kinase M2; Rag: Ras-related GTP-binding; S6K1: S6 kinase beta-1; SIRT1: Sirtuin 1; STING: Stimulator of interferon genes; TBK1: TANK-binding kinase 1; TRAF2: TNF receptor-associated factor 2; TFEB: Transcription factor EB; Th1/Th9: Type 1/9 T helper cell; TNF-α: Tumor necrosis factor-alpha; ULK1: Unc-51-like kinase 1; UPR: Unfolded protein response; VEGF: Vascular endothelial growth factor; VHL: Von Hippel-Lindau; ZAK: Leucine-zipper and sterile-α motif kinase

Within the TME, HIF-STING crosstalk exhibits complex regulatory patterns. In clear cell renal cell carcinoma (ccRCC), von Hippel-Lindau (VHL) deficiency upregulates IFN-β via HIF-2α, and BRCA1-associated protein 1 (BAP1) synergistically enhances STING and IFN-β transcriptional activity [131]. Notably, HIF-1α/2α elevation caused by VHL loss reduces mitochondrial membrane potential, promotes mtDNA release, activates cGAS-STING, and induces IFN-I production [132]. In liver cancer, hypoxia induces the DNA sensor DEAD-box helicase 41 (DDX41) via HIF-1, enhancing STING activation and promoting a senescence-associated secretory phenotype that recruits immune cells for tumor clearance [133]. In stress models such as T-2 toxin exposure, activation of the HIF-1α-cGAS-STING axis induces cellular senescence and apoptosis [134]. Similarly, selenoprotein W triggers PKM2 nuclear translocation and HIF-1α transcriptional activation, inducing mitochondrial damage, ROS generation, and mtDNA leakage—linking metabolic alterations to innate immune activation [135]. Fig. 5 illustrates the crosstalk between STING signaling and key energy regulators, including AMPK, mTOR, and HIF-1.

### MYC

MYC family proto-oncoproteins function as central transcription factors governing cell growth, metabolism, and proliferation, driving tumorigenesis and tumor progression through their integrated regulatory roles. MYC supports rapid cell growth and sustained proliferation by coordinating downstream target genes to enhance nutrient uptake, macromolecular biosynthesis, and ATP production [136, 137]. Emerging evidence has established MYC as a critical regulator of the cGAS-STING innate immune pathway, shaping the tumor immune microenvironment and therapeutic response through multilayered mechanisms [138].

In multiple cancer types, MYC negatively regulates STING signaling and contributes to immune-cold tumors. At the molecular level, c-MYC binds the STING enhancer region and represses its transcription, reducing expression of T-cell chemokines including CCL5, CXCL10, and CXCL11 [139]. N-MYC blocks STING signaling by inhibiting STING oligomerization and also attenuates RIG-I-like receptor (RLR) signaling by suppressing MAVS aggregation, thereby exerting dual inhibition on IFN-I induction [140]. This inhibitory signaling promotes immune-cold microenvironments, accelerates tumor progression, and induces therapeutic resistance in mantle cell lymphoma and triple-negative breast cancer—effects potentially involving MYC-dependent upregulation of CTP synthase 1 (CTPS1) and downregulation of STAT1 and T/NK cell chemokines [141].

Conversely, under inflammatory conditions such as acute lung injury, c-MYC forms a complex with cAMP response element-binding protein (CREB) that binds and activates the STING promoter to enhance transcription and promote inflammation [142]. Studies have also revealed that transgelin 2 (TAGLN2) recruits c-MYC and SRY-box transcription factor 9 (SOX9) to the Y-box binding protein 1 (YBX1) promoter and interacts directly with the AKT-YBX1 complex. This interaction promotes YBX1 phosphorylation and nuclear translocation, enhances single-stranded DNA accumulation, and activates the cGAS-STING pathway [143].

### p53

The canonical tumor-suppressive function of p53 arises from its ability to induce apoptosis, cell cycle arrest, and senescence. Recent studies have expanded this paradigm, identifying p53 as a central regulator of metabolic homeostasis. p53 maintains NAD⁺/NADH and ATP balance by suppressing glycolysis while enhancing OXPHOS and fatty acid oxidation. It also induces mitophagy to eliminate damaged organelles, preserving redox homeostasis [144, 145]. The crosstalk between p53 and the cGAS-STING pathway plays an essential role in genome stability, cell cycle surveillance, and anti-tumor immunity. Disruption of this axis contributes to tumorigenesis and immune escape.

p53 activates STING signaling through multiple mechanisms. It promotes degradation of the DNA exonuclease three prime repair exonuclease 1 (TREX1) by recruiting the ubiquitin ligase tripartite motif containing 24 (TRIM24), leading to cytoplasmic DNA accumulation that activates cGAS-STING and drives IFN-I production [146]. During the DNA damage response, p53 assembles a non-canonical STING signaling complex with Interferon gamma-inducible protein 16 (IFI16) and Ataxia telangiectasia mutated (ATM), where TRAF6-mediated K63-linked ubiquitination activates NF-κB and initiates STING-dependent gene expression [147]. Epigenetic factors modulate this network. For example, the long non-coding RNA NEAT1 attenuates anti-tumor immunity by repressing both p53 and cGAS-STING [148]. In p53-deficient backgrounds, STING modulation polarizes TAMs toward pro-inflammatory subtypes and induces apoptosis in gastric cancer cells via the IL6R-Janus Kinase (JAK)-IL24 axis, revealing context-dependent therapeutic opportunities [149].

Conversely, activated STING signaling exerts critical feedback regulation on p53. The second messenger cGAMP activates the DNA damage response in a STING-TBK1-dependent manner, where ATM autophosphorylation initiates the Checkpoint Kinase 2 (CHK2)-p53-p21 axis and triggers cell cycle arrest [150]. cGAMP also reduces intracellular NAD⁺ levels and inhibits poly ADP-ribose polymerase **(**PARP)-mediated homologous recombination repair [151]. However, STING overexpression promotes degradation of the upstream sensor IFI16 in osteosarcoma and non-small cell lung cancer (NSCLC) cells, inhibiting p53 transcriptional activity and pro-apoptotic function—revealing a tumor-promoting potential of STING under certain contexts [152]. Dysregulation of the p53-STING axis is linked to pathological conditions. In hypercholesterolemia models, elevated STING coincides with accumulation of DNA damage markers, including phosphorylated H2A histone family member X (γH2AX) and p53, suggesting that metabolic disturbance may compromise genome stability through this axis [153].

Cytoskeletal regulation also modulates this network upstream. Loss of the actin-related protein 2/3 (Arp2/3) complex activates the nuclear p53-p21 pathway and induces G1 arrest, while simultaneously activating cytoplasmic cGAS through micronucleus recognition to trigger STING-IRF3 interferon responses [154]. This network is critical for immune cell function. In T cells, cGAMP transport mediated by leucine rich repeat containing 8 VRAC subunit C (LRRC8C), a core component of the volume-regulated anion channel (VRAC), co-activates STING and p53, establishing an intrinsic inhibitory checkpoint that prevents excessive T cell activation [155, 156].

Mutant p53 targets components of the minichromosome maintenance (MCM) complex, increasing susceptibility to replication stress and chromosomal instability. The consequent constitutive elevation of cytoplasmic DNA sustains STING activation [157]. However, this aberrant activation fails to enhance anti-tumor immunity. Instead, it antagonizes canonical interferon responses and activates non-canonical NF-κB signaling to drive pro-tumor inflammatory gene expression. The resulting microenvironment becomes enriched with M2-like macrophages and exhibits an immune-cold phenotype that promotes resistance to immune checkpoint inhibitor therapy [158]. Conversely, reversing this chromosomal instability and depleting STING in cancer cells can eliminate the pro-metastatic effect of chromosomal instability (CIN) in an immunocompetent environment. Notably, whether CIN promotes or suppresses tumor progression depends on the interplay between the rate of CIN and the TME, particularly its immune components [159, 160].

### RAS

RAS-mediated signaling functions as a highly conserved transduction hub governing cell proliferation, differentiation, and survival through protein interactions and phosphorylation cascades [161]. As a key oncogene, RAS drives metabolic reprogramming by enhancing glycolysis, glucose uptake, autophagy, and lipid/nucleotide synthesis via the pentose phosphate pathway (PPP) [162, 163]. Emerging evidence reveals a tight interaction network between STING and oncogenic RAS signaling that collectively determines cell fate and tumor progression.

Oncogenic RAS isoforms, such as H-RASV12, act as critical triggers for cGAS-STING activation during early tumorigenesis. RAS induces DNA replication stress and damage, leading to micronucleus formation or leakage of cytoplasmic chromatin fragments. Recognition of abnormal DNA by cGAS initiates STING-dependent IFN-I responses. Nucleases, including meiotic recombination 11 homolog A (MRE11) and TREX1 fine-tune this process, representing an intrinsic surveillance mechanism against oncogene-induced genomic instability [164]. Conversely, activated STING signaling exerts multilayered feedback regulation on oncogenic RAS. STING-mediated inflammatory responses suppress activated RAS signaling, while IFN-β enhances sensitivity to ferroptosis in RAS-mutant cells, exerting tumor-suppressive effects [165]. Functional STING signaling relies on support from RAS superfamily members. For example, ubiquitination of the small GTPase secretion-associated Ras-related GTPase 1 A (SAR1A) facilitates STING trafficking from the ER to the Golgi apparatus, revealing deep integration of the two pathways at the level of fundamental cell biology [166].

The interplay between RAS and STING strongly influences cell fate decisions and tumor immune surveillance. The STING-generated second messenger 2’,3’-cGAMP modulates the small GTPase Rab18 and regulates Ras/Extracellular signal-regulated kinase **(**ERK) signaling to control cell migration [167]. Perturbation of stress-sensing nodes such as nuclear factor erythroid 2-related factor 2 (NRF2) affects both RAS network components and STING-associated kinase TBK1-mediated inflammatory responses. These pathways cooperate during stress responses to maintain cellular homeostasis [168]. Intact STING function is essential for immune surveillance and elimination of RAS-driven cells, whereas STING deficiency impairs this protective mechanism. Targeting the dynamic crosstalk between these pathways provides a rational basis for developing novel therapies against RAS-driven tumors.

### LKB1

Serine/threonine kinase LKB1 (STK11) functions as a key upstream regulator of cellular energy balance and metabolic homeostasis [169]. LKB1 directs cell growth, polarity, and tumor suppression by activating AMPK under energy stress, shifting metabolism from anabolism to catabolism for rapid ATP generation [170]. Loss of LKB1 disrupts AMPK regulation, drives metabolic reprogramming, and accelerates tumor growth [171].

LKB1 deficiency suppresses STING expression through coordinated epigenetic remodeling and organelle dysfunction. LKB1 inactivation elevates S-adenosylmethionine (SAM) levels and hyperactivates DNA methyltransferase 1 (DNMT1) and Enhancer of Zeste Homolog 2 (EZH2), which silence STING transcription via DNA hypermethylation and repressive histone modifications [172, 173]. Concurrently, LKB1 deficiency induces mitochondrial dysfunction and cytoplasmic accumulation of mitochondrial dsDNA. However, STING signaling remains insensitive to this dsDNA because its expression is epigenetically silenced, creating a state of immune blindness [174].

**Fig. 6:**
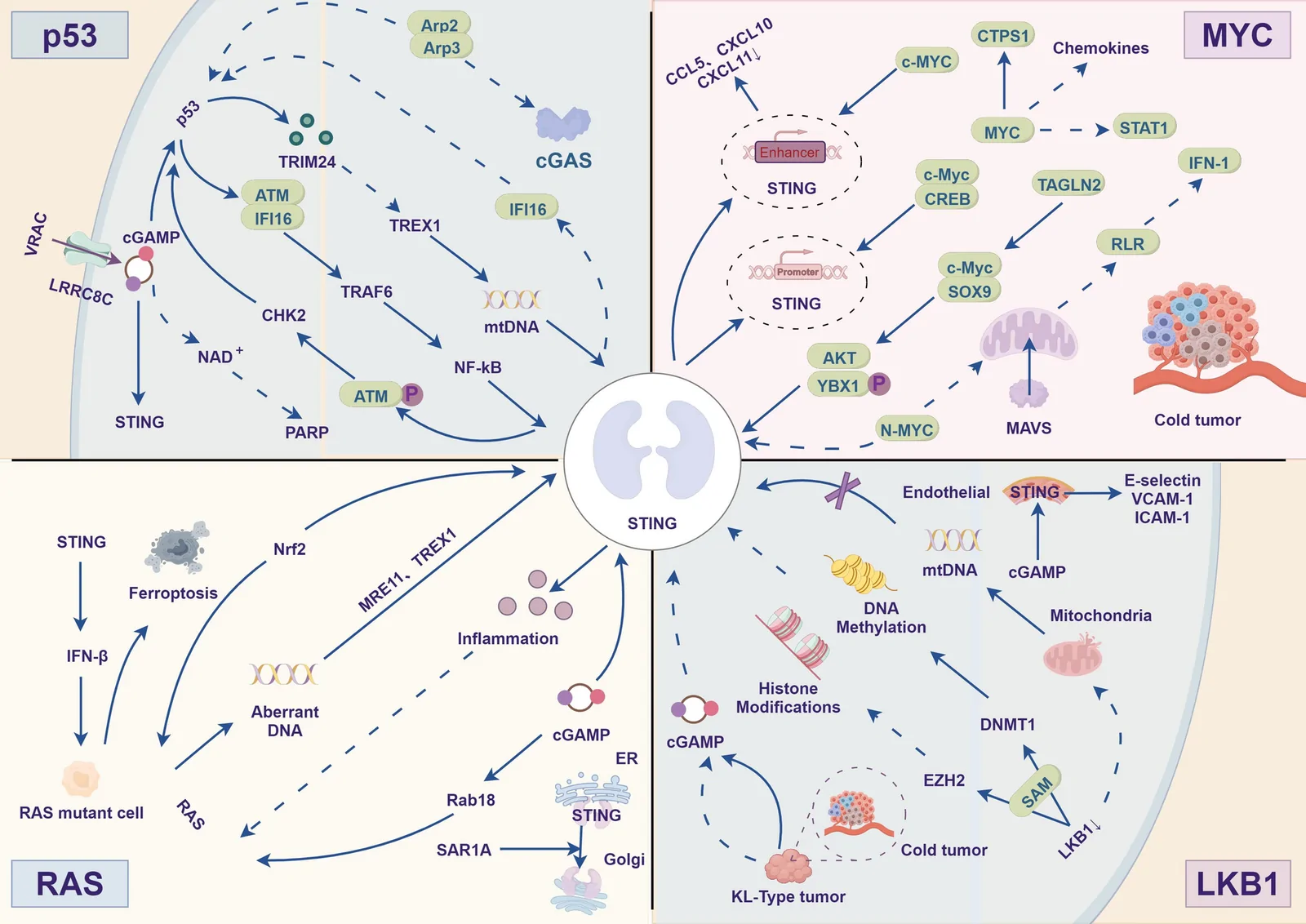
Crosstalk between STING Signaling and Key Energy Regulators, Including p53, MYC, RAS, and LKB1. AKT: RAC-alpha serine/threonine-protein kinase; Arp2/3: Actin-related protein 2/3; ATM: Ataxia telangiectasia mutated; cGAMP: Cyclic GMP-AMP; cGAS: Cyclic GMP-AMP synthase; CREB: cAMP response element-binding protein; CCL5: C-C motif chemokine ligand 5; CHK2: Checkpoint Kinase 2; CTPS1: CTP synthase 1; CXCL10: C-X-C motif chemokine ligand 10; DNMT1: DNA methyltransferase 1; ER: Endoplasmic reticulum; EZH2: Enhancer of zeste homolog 2; IFI16: Interferon gamma-inducible protein 16; IFN-I: Type I interferon; LKB1: Liver kinase B1; LRRC8C: leucine rich repeat containing 8 VRAC subunit C; MAVS: Mitochondrial antiviral signaling protein; MRE11: Meiotic recombination 11 homolog A; mtDNA: Mitochondrial DNA; MYC: Myelocytomatosis; NAD^+^: Nicotinamide adenine dinucleotide (Oxidized); NF-κB: Nuclear factor kappa-B; NRF2: Nuclear factor erythroid 2-related factor 2; PARP: Poly ADP-ribose polymerase; RAS: Ras-related GTP-binding; RLR: RIG-I-like receptor; SAM: S-adenosylmethionine; SAR1A: Secretion-associated Ras-related GTPase 1 A; SOX9: SRY-box transcription factor 9; STAT1: Signal transducer and activator of transcription 1; STING: Stimulator of interferon genes; TAGLN2: Transgelin 2; TRAF6: TNF receptor-associated factor 6; TREX1:Three prime repair exonuclease 1; VCAM-1: Vascular Cell Adhesion Molecule 1; VRAC: Volume-regulated anion channel; YBX1: Y-box binding protein 1

STING silencing represents a central mechanism underlying immune escape and immunotherapy resistance in tumors carrying concurrent KRAS and STK11 (LKB1) mutations—defined as KL-type tumors. While KRAS mutation alone impairs the IFN-I pathway at early stages, concurrent LKB1 deletion synergistically establishes a prominent immune-cold microenvironment, particularly in tumors with additional p53 mutations [175]. KL-type tumor cells maintain this immunosuppressive state through multiple strategies. They minimize intracellular accumulation of 2’,3’-cGAMP to prevent activation of the STING-STAT1 axis [176]. Paradoxically, they may also secrete cGAMP into the microenvironment via extracellular vesicles, activating STING signaling in tumor vascular endothelial cells. In this compartment, STING cooperates with IFN-I to increase vascular permeability and upregulate adhesion molecules, thereby promoting T-cell extravasation [177]. This finding reveals cell-type-specific and context-dependent roles of STING within the KL TME. Notably, while preclinical models indicate that STK11 mutation suppresses the STING-IFN-I pathway, clinical samples from STK11-deficient tumors often exhibit dysregulated activation of this pathway [178]. This discrepancy may arise from more complex cellular interactions and systemic effects operating within the patient’s TME. As shown in Fig. 6, STING signaling engages in crosstalk with key energy regulators, namely p53, MYC, RAS, and LKB1.

### Therapeutic potential of targeting the interplay between STING signaling and energy metabolic networks

The interplay between STING signaling and energy metabolic networks offers a conceptually new framework for cancer therapy. By targeting core metabolic pathways, including glycolysis, oxidative phosphorylation, lipid metabolism, and glutamine metabolism, mitochondrial stress and metabolic imbalance can be therapeutically induced, converting metabolic crises into STING-dependent immunogenic signals. At the molecular level, multiple strategies have shown promise in remodeling the immunosuppressive “cold” TME. These include modulating the AMPK/mTOR axis, reversing MYC-mediated transcriptional repression of STING, restoring STING expression epigenetically silenced in LKB1-deficient tumors, and leveraging the p53-STING axis to enhance chemo-radiotherapy sensitivity. Collectively, this section systematically delineates combinatorial intervention strategies centered on the STING-metabolism interface, highlighting their diverse translational potential in cancer immunotherapy.

### Combination strategies targeting STING signaling and core energy metabolic pathways

Mechanistic dissection of the crosstalk between STING signaling and core energy metabolic pathways has positioned the combined intervention of this network as a promising anti-tumor strategy. This approach employs pharmacological agents to target metabolic vulnerabilities in tumor cells, triggering intrinsic stress responses, including mitochondrial damage and metabolite imbalance, that activate the cGAS-STING pathway. By converting metabolic crisis into immunogenic signals, this strategy reverses immunosuppression and establishes durable anti-tumor immunity. Its implementation relies on advanced delivery technologies. Multifunctional nanocarriers, such as metal-organic frameworks and mesoporous silica, enable spatiotemporally synchronized delivery of STING agonists and metabolic modulators [179, 180]. Intelligent bioengineered systems, including engineered bacteria and near-infrared-responsive cell hydrogels, produce STING agonists or consume specific metabolites in situ, allowing on-demand, localized regulation [181, 182]. Table 1 summarizes strategies that combine targeted metabolic pathway intervention with STING activation in cancer therapy.

**Table 1:** Strategies combining targeted metabolic pathways with STING activation in cancer therapy

Targeted Metabolic Pathway	Cancer Type	Strategy	Core Mechanism of Action	Effect on STING Pathway	Main Immune Effect	References
Glycolysis	Hepatocellular Carcinoma	GO/CoF_2_: Integration of glucose oxidase (GOx) with cobalt fluoride (CoF_2_) nanozyme	Depletes glucose; generates ROS to induce pyroptosis; releases mtDNA	Activates (via mtDNA) and amplifies (via Co²⁺)	Triggers strong immune response; enhances efficacy of immune checkpoint inhibitors	[183]
Glycolysis	Hepatocellular Carcinoma	C-B-M-Mn²⁺: Encapsulation of Glut1 inhibitor BAY-876 and STING agonist MSA-2 in nanovesicle membranes	Inhibits glycolysis and increases ROS generation; inducing mtDNA leakage	Directly activates (MSA-2) and enhances (mtDNA)	Promotes DC maturation; recruits CD8⁺ T cells and NK cells; alleviates immune escape	[184]
Glycolysis	Hepatocellular Carcinoma	Mn-DCA-sora-MS: Multifunctional supramolecular microsphere based on cyclodextrin host-guest chemistry, loading sorafenib into porous microspheres co-mineralized with manganese and dichloroacetate (DCA)	Releases sorafenib for chemotherapy and embolization; DCA inhibits glycolysis-derived lactate	Activates (Mn²⁺, DCA)	Reverses immunosuppressive microenvironment; achieves local and systemic immune activation	[185]
Glycolysis	Orthotopic Liver Tumors	MnG: Manganese galvanic cell fabricated via liquid-phase exfoliation and in situ electrochemical replacement	Sustained release of hydrogen gas and Mn²⁺; regulates glucose metabolism to inhibit TREX2	Activates and amplifies (Mn²⁺, inhibits TREX2)	Elicits potent anti-tumor immune response; enhances efficacy of immune checkpoint inhibitors	[186]
Glycolysis	Breast Cancer	LT@MnO@MON-HA: Nanocarrier based on MnO nanoparticles and mesoporous silica shell, loaded with Lonidamine, coated with hyaluronic acid	Disrupts mitochondrial metabolism and elevates ROS; catalyzes hydroxyl radical generation; depletes glutathione	Activates (Mn²⁺, mitochondrial damage)	Stimulates cytokine release; activates effector T cells; induces systemic immune response	[180]
Glycolysis	Breast Cancer	Cu-MOF@MnO₂/GOx: Cu-based metal-organic framework sequentially loaded with MnO₂ and GOx	Depletes glucose; enhances susceptibility to cuproptosis; alleviates hypoxia; induces cuproptosis	Activates and amplifies (Mn²⁺, mtDNA)	Activates innate and adaptive immune responses; enhances efficacy of immune checkpoint inhibitors	[179]
Glycolysis	Breast Cancer	DB@CSCN: Genetically programmed *E. coli* producing STING agonist cyclic diadenylate (CDA), functionalized with copper‑sulfur nanoparticles	Hypoxia-targeted tumor delivery of engineered bacteria; induces cuproptosis; disrupts glycolysis; releases CDA to activate STING	Directly activates (CDA) and enhances (via glycolysis disruption)	Promotes DC maturation and T cell immunity; inhibits tumor proliferation and metastasis	[181]
Glycolysis	Colorectal Cancer	AND-gated living hydrogel: Encapsulation of genetically engineered bacteria expressing lactate oxidase and glucose oxidase in a NIR-light responsive hydrogel	Laser-triggers engineered bacteria to express lactate/glucose oxidase; weakens glycolysis; improves mitochondrial respiration	Activates	Enhances T cell response; polarizes M1 macrophages; induces ICD	[182]
Glycolysis	Melanoma	PtIrFeMoZn: High-entropy alloy nanozyme synthesized via one-step hydrothermal method, treatable with alternating current	Catalyzes generation of a ROS storm; induces apoptosis and ferroptosis; generates hypochlorous acid; consumes NADH to disrupt glycolytic balance	Activates	Enhances ICD; activates anti-tumor immune response	[187]
Glycolysis/OXPHOS	Breast Cancer	Zn-PEN: Fibrous therapeutic nanoformulation prepared by chelating the copper chelator D-penicillamine with Zn²⁺	Copper chelation and depletion, releases Zn²⁺; inhibits OXPHOS and glycolysis; clears Fusobacterium nucleatum; inhibits epithelial-mesenchymal transition	Activates (Zn²⁺)	Induces tumor cell death; alleviates immunosuppression; inhibits tumor metastasis	[188]
Glycolysis/OXPHOS	Glioblastoma	HFn/ZIF-8@L820/TP5: Co-encapsulation of Lonidamine-IR820 conjugate (L820) and immune modulator TP5 in ZIF-8 framework, surface-coated with H-ferritin	Triggers mitochondrial membrane potential collapse; ATP depletion; AMPK activation; inhibits GLUT1/HIF-1α impairing glycolysis	Activates (mtDNA release)	Promotes ICD; enhances T cell activity and suppresses regulatory T cells; remodels immune microenvironment	[189]
OXPHOS	Cervical Cancer	MCSP: Polyethylene glycolylated manganese-doped calcium sulfide nanoparticles for gas-amplified metal immunotherapy	Interferes with OXPHOS pathway; disrupts calcium buffering causing pyroptosis and mitochondrial damage	Enhances activation (Mn²⁺, mtDNA release)	Activates DC; synergizes with PD-1 therapy to enhance anti-tumor immunity	[190]
Lipid Metabolism	Breast Cancer	MMONs-irisin: Nanomaterial platform for co-delivery of irisin and manganese ions, doped in mesoporous organosilica nanoparticles	Promotes lipolysis; inhibits lipogenesis; depletes intratumoral lipid droplets	Activates (via lipid droplet depletion, Mn²⁺)	Enhances DC antigen presentation and CD8⁺ T cell cytotoxicity; reverses immunosuppression; sensitizes to anti-PD-1 therapy	[191]
Lipid Metabolism	Non-Small Cell Lung Cancer	¹³¹I-Mn/SAE@M: Multi-enzyme catalytic radiopharmaceutical using tumor cell membrane-coated Mn single-atom nanoenzyme carrier to target and deliver Iodine-131 (¹³¹I)	Generates ROS and alleviates hypoxia; disrupts lipid metabolic homeostasis; inducing ferroptosis	Activates	Elicits systemic anti-tumor immunity and immune memory	[192]
Tyrosine/Lipid Metabolism	Breast Cancer	ITCC NPs: Multifunctional phototheranostic nanoplatform based on A-D-A-type photovoltaic molecules, self-assembled into water-soluble nanoparticles with DSPE-PEG-NH₂-2000 assistance	Generates ROS and heat; promotes vascular normalization; induces tyrosine and lipid metabolism reprogramming	Activates	Induces ICD; promotes activation and infiltration of DCs, NK cells, and T cells; suppresses Tregs; remodels immune microenvironment	[193]
Glutamine Metabolism	Colorectal Cancer	HA/E-M@Purpurin NPs: Dual-targeting nano-herb based on manganese coordination, combining glutamine regulation and tumor stem cell trait inhibition	Inhibits glutaminolysis; causes compensatory glutamine accumulation	Activates (Mn²⁺)	Efficiently activates DCs; inhibits stem cell-like traits of tumor cells	[194]
Glutamine Metabolism	Colorectal Cancer	MSR‑CPT/APPs: Oncolytic magnetotactic bacteria loaded with camptothecin and anti‑PD‑L1 peptide, were further encapsulated with mulberry leaf lipids and Pluronic F127	Elevates the levels of beneficial metabolites (short‑chain fatty acids and citrulline); reduces the levels of harmful metabolites (L‑glutamine and kynurenic acid)	Activates	Induces ICD; promotes macrophage polarization toward the M1 phenotype; enhances the secretion of pro‑inflammatory cytokines; prolongs the duration of T‑cell recruitment, and reduces the proportion of immunosuppressive cells	[195]
Glutamine Metabolism	Breast Cancer	HM‑BPT: A functional biomimetic nanoplatform consisting of manganese oxide (MnO₂) nanoparticles coated with mesenchymal stem cell membranes (MSCm) and pH‑sensitive liposomes (pSL), designed for the delivery of the glutamine metabolism inhibitor BPTES	Supplies oxygen and depletes glutathione (GSH)	Activates	Promotes the infiltration of CTLs and induces the polarization of macrophages to the M1 phenotype	[196]

Interference with glycolysis, such as inhibiting glucose uptake, blocks tumor energy supply and induces mitochondrial ROS bursts and mtDNA leakage, providing critical signals for cGAS-STING activation [183, 184]. Combining the cyclooxygenase-2 (COX-2) inhibitor celecoxib with cGAMP synergistically enhances anti-tumor immunity by regulating glycolysis-related genes and reducing lactate efflux [197]. Direct disruption of OXPHOS, such as impairing mitochondrial membrane potential or inhibiting IDH3α, effectively triggers mitochondrial stress and mtDNA release. Tumors with activated STING signaling show enrichment of OXPHOS and DNA repair pathways, revealing intrinsic links between metabolism and DNA damage repair [198]. Accordingly, the PARP inhibitor talazoparib activates STING by trapping PARP1 on chromatin and inducing DNA damage [198].

In lipid metabolism, promoting lipolysis or depleting lipid droplets induces lipotoxic stress, indirectly activating STING through mitochondrial damage and triggering anti-tumor neutrophil infiltration in triple-negative breast cancer models [76, 191–193]. Targeted inhibition of glutamine metabolism compromises tumor biosynthesis, and the resulting metabolic stress can be converted into immunological recognition signals [194–196]. The bifunctional prodrug T26 depletes extracellular glutamine while directly activating innate immunity, generating synergistic anti-tumor effects [199]. Manganese ions are widely applicable in these strategies, serving as direct STING agonists while also catalyzing ROS production and inhibiting DNA-degrading enzymes—providing dual enhancement of STING signaling [185, 186].

This combinatorial strategy induces DC activation and maturation, enhances antigen presentation, and establishes a foundation for adaptive immune responses [190]. It promotes effector T cell infiltration, particularly CD8⁺ T cells, while suppressing Treg-mediated immunoregulation [180, 189]. Furthermore, it reshapes the immunosuppressive microenvironment by driving macrophage polarization toward M1 phenotype and exhibits strong synergy with immune checkpoint inhibitors [182]. Notably, many metabolic intervention agents induce immunogenic cell death (ICD), including pyroptosis and ferroptosis, releasing tumor antigens and danger signals that cooperate with STING-derived interferons to trigger robust systemic anti-tumor responses and support long-term immune memory [187, 188].

It must be emphasized that STING-mediated metabolic regulation acts as a double-edged sword: while STING activation exerts anti-tumor effects, excessive activation exacerbates pathological progression in metabolic diseases and fibrosis. Therefore, STING inhibition strategies are equally critical in specific contexts. In pulmonary fibrosis, targeting the upstream lipid metabolic enzyme PLA2G2A or its downstream effector IDO1 blocks STING-associated profibrotic signaling [89]. For abnormally activated STING in metabolic diseases, interventions include LXR agonists (which induce the cGAMP-degrading enzyme SMPDL3A), fatty acid synthase inhibitors (which alleviate lipid metabolic stress), or NRF2 activators such as S217879 (which exert anti-steatotic effects and attenuate inflammation in metabolic-associated fatty liver disease models) [83, 86, 200]. These findings also inform strategies for suppressing chronic inflammation driven by aberrant STING activation within the TME.

In summary, the core logic of these strategies involves inducing cellular stress through metabolic intervention, activating STING, reshaping immune responses, and generating a cascade reaction. The critical step lies in converting tumor metabolic vulnerabilities into actionable immunogenic signals. Future directions include developing smarter delivery systems, exploring multi-pathway combinatorial regimens, and designing personalized strategies based on individual metabolic and genetic profiles. Such approaches can reverse immunosuppressive TME into immune-active states and improve clinical outcomes. Furthermore, a deeper understanding of the bidirectional regulatory mechanisms of STING in metabolic homeostasis will provide novel therapeutic perspectives for fibrosis, metabolic diseases, and beyond.

### Combination strategies targeting STING signaling and key energy metabolic regulators

The regulatory networks formed between STING signaling and core energy-sensing molecules provide a central framework for understanding metabolic-immune crosstalk within the TME. Targeting key nodal intersections, such as suppressing the AMPKα1-STING positive feedback loop, reversing MYC-mediated STING inhibition, or modulating p53 and LKB1, establishes a foundation for developing novel combinatorial therapies.

AMPK and mTOR serve as central regulators of cellular energy sensing, maintaining homeostasis through opposing actions while directly interacting with STING. In NSCLC, TAMs drive M2 polarization via an AMPKα1-STING positive feedback loop, promoting tumor cell migration. Ginsenosides impede this loop, blocking M2 polarization and epithelial-mesenchymal transition [201]. Similarly, impaired AMPK signaling in gelatin-treated U937 cells induces mitochondrial fission and dysfunction, leading to cytoplasmic mtDNA leakage and STING activation, while enhanced glycolysis further elevates cellular phagocytic activity [202]. The ZAK inhibitor iZAK2, alone or with STING agonists or anti-PD-1, suppresses ZAK-AKT-mTOR signaling to inhibit tumor growth in lung cancer models [118].

Targeting strategies hold particular importance in hypoxic conditions. The TREM2-targeted STING agonist prodrug GB2 reprograms macrophages through dual mechanisms: upregulating the glycolysis-ROS-HIF-1α axis to enhance glycolytic metabolism and inflammatory cytokine expression, while inducing mitochondria-ER contacts to strengthen Ca²⁺-mediated phagocytosis. These effects revert macrophages to M1 phenotype and promote CD8⁺ T-cell infiltration. Preclinical models demonstrate synergistic antitumor effects of GB2 alone or with anti-PD-1 agents [130]. Paclitaxel efficacy is superior in STING wild-type hepatocellular carcinoma compared with STING-knockout models, indicating dependence on intact STING signaling. Paclitaxel induces cytoplasmic DNA accumulation, activates the DNA sensor DDX41, initiates STING signaling, and triggers a senescence-associated secretory phenotype. Hypoxia upregulates DDX41 via HIF-1, sensitizing tumors to this pathway, and the senescence-associated secretory phenotype (SASP)-mediated immune recruitment promotes tumor clearance [133].

High MYC expression correlates with poor prognosis in head and neck squamous cell carcinoma [203], high-grade serous ovarian cancer [140], and primary metastatic neuroblastoma [204], associating with reduced interferon pathway activity and diminished immune infiltration. Genetic or pharmacological inhibition of MYC relieves STING transcriptional repression, activates cGAS-STING-IRF3 through DNA damage responses, and promotes chemokine production and CD8⁺ T-cell recruitment [203]. Targeting upstream nodes also shows promise: lysine acetyltransferase 6 A (KAT6A) inhibitors alleviate c-MYC/DNMT1-mediated epigenetic silencing of cGAS, while disruption of the TAGLN2-YBX1-AKT complex reduces abnormal DNA accumulation and restores STING function [205].

The p53-STING axis is a key therapeutic target. Clomiphene directly induces p53-STING interaction, activates non-canonical NF-κB signaling, and enhances T-cell immunity [206]. The PARP inhibitor talazoparib induces senescence by suppressing p53 ubiquitination; combination with the CDK4/6 inhibitor palbociclib synergistically activates cGAS-STING, and SASP induction reshapes the TME by increasing CD8⁺ T-cell and NK cell infiltration while reducing M2 macrophages [207]. For chemosensitization, cGAMP upregulates p53 by inhibiting Phosphoinositide 3-Kinase (PI3K)/AKT signaling and enhances oxaliplatin sensitivity in drug-resistant tumors [208]. In radiotherapy, the Wnt/β-catenin inhibitor ICG-001 increases radiation-induced DNA damage by suppressing p53, activating cGAS-STING, and enhancing radiosensitivity and long-term immune memory [209]. The DNA-PK inhibitor peposertib selectively enhances radiosensitivity in p53-deficient backgrounds, where micronucleus formation activates STING-dependent immune responses and upregulates PD-L1, achieving combined cytotoxic and immunoregulatory effects [210].

Strategies targeting RAS-mutant tumors enhance immunotherapy efficacy through innate immune activation. In KRAS-mutant lung cancer, cisplatin activates cGAS-STING via DNA damage and synergizes with PD-1 antibodies to promote CD8⁺ T-cell infiltration [211]. In H-RAS-driven head and neck cancer models, local STING agonist combined with PD-1/Cytotoxic T-Lymphocyte-Associated Protein 4 (CTLA-4) blockade activates IFN-I signaling and reverses immune resistance [212]. In pancreatic cancer, lipid nanoparticles co-delivering STING and TLR4 agonists combined with RAS-targeted therapy reprogram the immunosuppressive microenvironment and elicit robust T-cell immunity [213]. Lipid nanoparticles co-delivering cGAMP and KRAS G12D neoantigen-encoding mRNA activate CD8⁺ cytotoxic T cells, generating durable anti-tumor immunity. These strategies aim to convert RAS-mutant immune-cold tumors into immunotherapy-sensitive hot tumors through STING activation [214].

Immunotherapy resistance in LKB1-mutant lung cancer arises from multiple mechanisms. Tumor cells suppress STING signaling through formation of an LKB1-IAP-JAK1 complex. In KRAS-LKB1 co-mutant subtypes, mitochondrial dysfunction drives epigenetic STING silencing, generating immune-cold tumors [215, 216]. Current strategies focus on multi-level immune activation: IAP inhibitors restore STING signaling [215]; decitabine combined with MPS1 inhibitor relieves epigenetic repression [176]; metformin suppresses colony formation of LKB1-mutant cells while modulating STING signaling in immune cells to promote antitumor conversion [217]. Table 2 summarizes the functional status of STING signaling across various cancer types, the underlying metabolic crosstalk, and the corresponding therapeutic outcomes.

**Table 2:** STING functional status and therapeutic outcomes across cancer types

Cancer Type	STING Functional Status	Key Metabolic Crosstalk	STING-Related Therapeutic Strategy	Outcome / Immune Effect	References
Non-small cell lung cancer	Chronically activated (pro-tumorigenic)	AMPKα1/STING positive feedback loop drives macrophage M2 polarization and tumor cell migration	Ginsenosides (inhibit AMPKα1/STING loop)	Blocks M2 macrophage polarization and epithelial-mesenchymal transition	[201]
Lung squamous cell carcinoma	Alternatively activated (DNA-PK-mediated)	DNA-PK activates ZAK-AKT-mTOR signaling; promotes glycolysis and chemoresistance	ZAK inhibitor ± STING agonist ± anti-PD-1 antibody	Suppresses tumor growth in mouse models and patient-derived xenograft	[118]
Colorectal cancer	Suppressed (context-dependent)	TREM2 overexpression in TAMs; STING pathway downregulated	TREM2-targeted STING prodrug (GB2)	Induces tumor regression; reverses immunosuppression; promotes CD8⁺ T infiltration	[130]
Hepatocellular carcinoma	Chronically activated (double-edged)	Hypoxia → HIF-1α → DDX41 → STING activation → SASP; hypoxia sensitizes the pathway	Paclitaxel (exploits the hypoxia-induced DDX41/STING axis)	SASP recruits immune cells for tumor clearance; risk of chronic inflammation	[133]
Head and neck / Ovarian / Neuroblastoma	Suppressed (transcriptional)	MYC binds STING enhancer → represses transcription; decreased chemokine expression	MYC inhibition (genetic or pharmacological)	Reactivates cGAS-STING-IRF3; increases CD8⁺ T cell chemokines	[140, 203, 204]
Colorectal cancer	Suppressed (epigenetic)	MYC/DNMT1-mediated cGAS silencing; impaired interferon signaling	KAT6A inhibition (relieves c-MYC/DNMT1-mediated repression)	Enhances CD8⁺ T cell infiltration via IFN-I activation	[180]
Gastric cancer	Hyperactivated (pro-resistance)	TAGLN2 enhances YBX1-related single-stranded DNA accumulation → cGAS-STING activation → upregulates ISGs	Fisetin or MK2206 (inhibit TAGLN2-YBX1-AKT axis)	Reduces single-stranded DNA accumulation, ISG upregulation, and therapy resistance	[143]
Melanoma / Lung cancer	Suppressed (immune evasion)	STING signaling inhibited; immunosuppressive microenvironment established	Clofarabine (induces p53-STING-NF-κB non-canonical pathway)	Induces apoptosis, GSDME-related pyroptosis, and CD8⁺ T cell anti-tumor activity	[206]
Colorectal cancer	Suppressed (immune evasion)	STING signaling inhibited; impaired senescence-associated immune surveillance	Talazoparib (PARP inhibitor) + Palbociclib (CDK4/6 inhibitor)	Induces cGAS/STING activation and SASP; increases CD8⁺ T and NK cells; reduces M2 macrophages and MDSCs	[207]
Hepatocellular carcinoma	Suppressed (Wnt/β-catenin-mediated)	Wnt/β-catenin activation induces radioresistance; inhibits STING pathway	ICG-001 (Wnt/β-catenin inhibitor) + radiotherapy	Enhances radio sensitivity and long-term immune memory via p53-STING axis	[209]
Pancreatic ductal adenocarcinoma	Suppressed (immune tolerance)	KRAS mutation + hepatic immune tolerance; STING signaling impaired	Lipid nanoparticle co-delivering STING + TLR4 agonists + RAS-targeted therapy	Reprograms immunosuppressive TME; enhances anti-tumor immunity	[213]
Pancreatic ductal adenocarcinoma	Suppressed (immune tolerance)	KRAS mutation; impaired antigen presentation and T cell priming	Lipid nanoparticle delivering STING agonist (cGAMP) + KRAS G12D neoantigen mRNA	Activates perforin-releasing CD8⁺ cytotoxic T cells; generates durable anti-tumor immunity	[214]
LKB1-mutant lung cancer	Silenced (immune evasion)	LKB1 loss leads to immune evasion and therapy resistance	IAP inhibitors (restore JAK1-regulated STING expression)	Restores DNA sensing; enhances cytotoxic immune cell infiltration; anti-tumor activity in immune-competent models	[215]
KRAS-LKB1 (KL) co-mutant lung cancer	Silenced (epigenetic)	LKB1 loss → DNMT1/EZH2-mediated STING hypermethylation; reduced 2′3′-cGAMP accumulation to avoid STING/STAT1 activation	Decitabine (priming) + pulsed MPS1 inhibitor	Epigenetic derepression of STING; restores T cell infiltration; enhances anti-PD-1 efficacy; durable remission	[216]
Non-small cell lung cancer	Activatable (metformin-sensitive)	LKB1-mutant cells exhibit metabolic vulnerability; STING pathway intact but suppressed	Metformin (activates cGAS-STING in PBMCs) + immunotherapy	Synergistic effect; significantly reduces colony formation of LKB1-mutant NSCLC cells	[217]

**Fig. 7:**
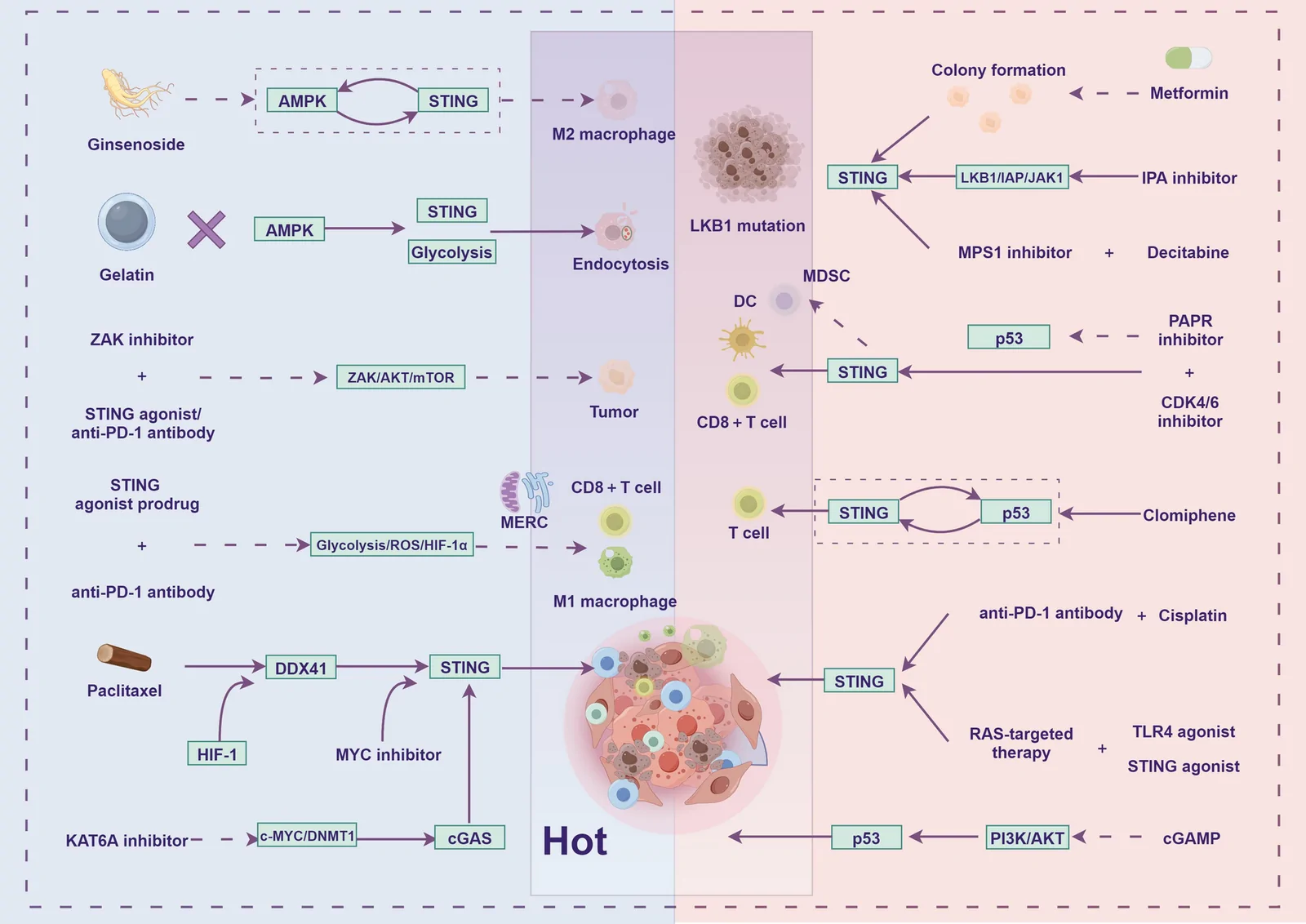
Combination strategies targeting STING signaling and key energy metabolic regulators. AKT: RAC-alpha serine/threonine-protein kinase; AMPK: AMP-activated protein kinase; cGAMP: Cyclic GMP-AMP; cGAS: Cyclic GMP-AMP synthase; DC: Dendritic cell; DDX41: DNA sensor DEAD-box helicase 41; HIF-1: Hypoxia-inducible factor 1; JAK1: Janus Kinase 1; LKB1: Liver kinase B1; MDSC: Myeloid-derived suppressor cell; MERC: Mitochondria-endoplasmic reticulum contact; MYC: Myelocytomatosis; PARP: Poly ADP-ribose polymerase 1; PI3K: Phosphoinositide 3-Kinase; RAS: Rat sarcoma; ROS: Reactive oxygen species; STING: Stimulator of interferon genes; ZAK: Leucine-zipper and sterile-α motif kinase

In summary, STING signaling forms an interactive network with core energy-regulatory molecules, including AMPK/mTOR, MYC, LKB1, and p53, within the TME, coupling metabolic adaptation with immune remodeling (Fig. 7). From an energetic perspective, tumor and immune cells coordinate survival and function through shared nodes: AMPK/mTOR balance, MYC-driven metabolic reprogramming, LKB1-related mitochondrial function, and p53-mediated stress responses. Notably, STING-silent tumors are inherently resistant to STING agonists and exhibit an “immune-cold” phenotype, necessitating “priming” strategies (such as DNMT inhibitors) to reverse this silencing. Conversely, while STING-active tumors may directly respond to STING agonist-based therapy, the risk of chronic inflammation or T cell exhaustion resulting from excessive activation should be carefully monitored. Therefore, future efforts should focus on patient stratification based on STING expression status and the development of innovative combination regimens that co-modulate metabolism and immunity, aiming to more effectively convert “cold” tumors into immunotherapy-sensitive “hot” tumors.

### Implications and conclusions

The cGAS-STING signaling pathway is a central molecular hub that maintains cellular energy metabolic homeostasis and orchestrates innate immune responses. Its biological functions within the TME extend well beyond canonical cytoplasmic DNA sensing, operating as a precise monitor and dynamic responder to disruptions in energy homeostasis. This review systematically delineates how dysregulated energy metabolism in the TME engages the cGAS-STING cascade through two primary layers: aberrant activity in core metabolic pathways—including glycolysis, oxidative phosphorylation, lipid metabolism, and glutaminolysis—and dysregulation of key energy regulators such as AMPK, mTOR, HIF, MYC, p53, RAS, and LKB1. These perturbations trigger cytoplasmic leakage of mtDNA, abnormal accumulation of metabolic intermediates, and DNA replication stress.

STING signaling serves as a core transmitter of metabolic stress. It reshapes the metabolic adaptability of tumor cells and infiltrating immune cells and transduces energy imbalance into immune recognition signals, ultimately governing the transition of the TME from immune surveillance to immune escape. Acute, high-intensity activation (e.g., radiotherapy or high-dose STING agonists) can directly induce tumor cell death through transient ROS bursts and mitochondrial collapse, while also triggering ICD and STING-dependent adaptive immunity. In contrast, sustained STING activation resulting from metabolic dysfunction disrupts systemic metabolic homeostasis, drives the formation of an immunosuppressive microenvironment, and thereby promotes tumor immune evasion and distant metastasis. In severe cases, it may further trigger systemic metabolic dysregulation and cytokine storm-associated cachexia.

Such functional heterogeneity, rooted in energy homeostasis, suggests that precision STING-targeted interventions must move beyond the binary paradigm of complete activation or inhibition. Strategies should instead consider thresholds of metabolic imbalance, nonlinear relationships between signal intensity and duration, and cell-type-specific metabolic response programs. Current combination regimens centered on STING agonists hold substantial translational promise. Metabolism-targeting interventions, such as inhibition of the glycolytic rate-limiting enzyme PFKFB3 to exacerbate energy crisis, or glutaminase blockade to curtail anabolic substrate supply, can be integrated with epigenetic modulation. For instance, DNA methyltransferase inhibitors reverse STING silencing mediated by LKB1 mutations. These approaches help restore local energy homeostasis and effectively reverse immune-cold TME.

Nevertheless, clinical translation faces three major bottlenecks. First, species-specific differences in binding affinity and conformational responses to cyclic dinucleotide (CDN) agonists between human and murine STING significantly reduce the predictive value of preclinical models. Second, the metabolic–immune threshold paradox reflects nonlinear relationships between energy imbalance and STING-mediated immune outcomes: moderate imbalance may activate immune surveillance, whereas severe or sustained disturbance can lead to immune tolerance or exhaustion, complicating the definition of a safe therapeutic window. Third, spatiotemporal delivery limitations arise from systemic administration, which risks disrupting whole-body metabolic homeostasis and inducing non-specific inflammation or cytokine storms, imposing stringent requirements for targeted and controlled release.

Future research must integrate advanced paradigms from systems biology and synthetic immunology to dissect the spatiotemporal dynamics and molecular logic of regulatory networks centered on energy homeostasis. Key breakthroughs are anticipated in three directions. First, elucidating the spatiotemporal dynamics and cell-type specificity of energy network dysregulation will be essential. Single-cell metabolomics, spatial transcriptomics, and high-resolution in vivo imaging should clarify how distinct cell subsets undergo divergent trajectories of metabolic imbalance under specific TME stresses, and how STING signaling couples these events to global immune remodeling. Emphasis should be placed on molecular mechanisms of mtDNA release, crosstalk between metabolite accumulation and epigenetic modifications, and the central role of organelle dynamics.

Second, the development of spatiotemporally precise tools for metabolic control is critical. Smart delivery systems responsive to endogenous TME metabolic signals, such as lactate, ATP/AMP ratio, and pH gradients, or exogenous physical triggers including light, heat, and magnetic fields, will enable on-demand, site-specific, and temporally controlled co-delivery of STING agonists and metabolic modulators. Examples include pH-responsive nanoparticles, enzyme-sensitive liposomes, and synthetically engineered cellular carriers such as mesenchymal stem cells or attenuated Salmonella. Parallel efforts should focus on screening and optimizing small molecules that restore homeostasis by targeting core energy regulators, such as AMPK agonists and selective mTORC1 inhibitors. Biological agents that modulate post-translational modifications of STING, including palmitoylation inhibitors and SUMOylation regulators, may allow fine-tuning of signaling output thresholds.

Third, establishing multi-omics-guided personalized immuno-metabolic combination therapies will be pivotal. Future clinical trials should prospectively integrate multidimensional molecular profiles of patients, with particular attention to the therapeutic potential of predictive metabolic biomarkers. These include genetic backgrounds (e.g., concurrent KRAS and STK11/LKB1 mutations, TP53 mutation status), metabolic pathway signatures (e.g., glycolysis-dominant versus oxidative phosphorylation-dominant), and functional metabolic assays (e.g., NAD⁺/NADH ratio in tumor biopsy tissues). Such biomarkers can be used to define “STING-responsive” metabolic subtypes, thereby enabling adaptive clinical trial designs that calibrate the thresholds and dynamics of energy imbalance, redirecting STING signaling from pathological chronic activation toward physiological immune stimulation. This will drive a paradigm shift from empirical combination regimens to mechanism-driven precision immuno-metabolic therapy. However, translating these concepts into clinical practice will still require systematic validation through future prospective clinical studies.

Only by applying the systematic principles of energy homeostasis regulation across the full discovery-to-clinical translation continuum can we unravel the complex network of energy competition and metabolic dependence between tumors and the immune system. Precise decoding and manipulation of the cGAS-STING metabolic-immune hub will provide a robust theoretical foundation and actionable precision strategies for the next generation of cancer immunotherapy.

## Data Availability

No datasets were generated or analysed during the current study.

## Abbreviations

ACC: Acetyl-CoA carboxylase
AHRR: Aryl hydrocarbon receptor repressor
AKT: RAC-alpha serine/threonine-protein kinase
AMPK: AMP-activated protein kinase
ARF: ADP-ribosylation factor
Arp2/3: Actin-related protein 2/3
ATM: Ataxia telangiectasia mutated
ATG16L1: Autophagy-related 16-like 1
AUP1: Ancient ubiquitous protein 1
BAP1: BRCA1-associated protein 1
BATF2: Basic leucine zipper ATF-like transcription factor 2
CAFs: Cancer-associated fibroblasts
CAMKK2: Calcium/calmodulin-dependent protein kinase kinase 2
CDK4/6: Cyclin-Dependent Kinase 4/6
CDN: Cyclic dinucleotide
cGAMP: Cyclic GMP-AMP
cGAS: Cyclic GMP-AMP synthase
COPⅠ/Ⅱ: Coat protein complex Ⅰ/Ⅱ
COX-2: Cyclooxygenase-2
CRAT: Carnitine acetyltransferase
CREB: cAMP response element-binding protein
CTLA-4: Cytotoxic T-Lymphocyte-Associated Protein 4
CPT1A: Carnitine palmitoyltransferase 1 A
CTLs: Cytotoxic T lymphocytes
CTPS1: CTP synthase 1
DCs: Dendritic cells
DDX41: DNA sensor DEAD-box helicase 41
DNA-PK: DNA-dependent protein kinase
DNMT1: DNA methyltransferase 1
DUOX2: Dual oxidase 2
dsDNA: Double-stranded DNA
ER: Endoplasmic reticulum
ERK: Extracellular signal-regulated kinase
ETC: Electron transport chain
EZH2: Enhancer of zeste homolog 2
FAO: Fatty acid β-oxidation
FAD: Flavin adenine dinucleotide
FADH: Flavin adenine dinucleotide hydrate
FASN: Fatty acid synthase
FBP1: Fructose-1,6-bisphosphatase 1
FGF21: Fibroblast growth factor 21
FLCN: Folliculin
FNIP: Folliculin-interacting protein
GABARAP: Gamma-aminobutyric acid type A receptor-associated protein
GLUT1: Glucose transporter type 1
GSH: Glutathione
HIF-1α: Hypoxia-inducible factor 1-alpha
HK2: Hexokinase 2
ICD: Immunogenic cell death
ICIs: Immune checkpoint inhibitors
IDO1: Indoleamine 2,3-dioxygenase 1
IFI16: Interferon gamma-inducible protein 16
IFN-I: Type I interferon
IKK: IκB kinase
IMM: Inner mitochondrial membrane
IRE1α: Inositol-requiring enzyme 1α
IRF3: Interferon regulatory factor 3
ISGs: Interferon-stimulated genes
JAK: Janus Kinase
JNK: c-Jun N-terminal kinase
LKB1: Liver kinase B1
LXR: Liver X receptor
MARCH1: Membrane-associated RING-CH protein 1
MAVS: Mitochondrial antiviral signaling protein
MCM: Minichromosome maintenance
MRE11: Meiotic recombination 11 homolog A
mTORC: Mammalian target of rapamycin complex
mtDNA: Mitochondrial DNA
MYC: Myelocytomatosis
NAD⁺: Nicotinamide adenine dinucleotide (Oxidized)
NADH: Nicotinamide adenine dinucleotide (Hydride)
NAMPT: Nicotinamide phosphoribosyltransferase
NETs: Neutrophil extracellular traps
NF-κB: Nuclear factor kappa-B
NK: Natural killer
NLRP3: NOD-like receptor family pyrin domain containing 3
NRF2: Nuclear factor erythroid 2-related factor 2
OXPHOS: Oxidative phosphorylation
PARP-1: Poly ADP-ribose polymerase 1
PFKFB3: 6-Phosphofructo-2-kinase/fructose-2,6-bisphosphatase 3
PGC1α: Peroxisome proliferator-activated receptor γ coactivator 1-α
PI3K: Phosphoinositide 3-kinase
PKM2: Pyruvate kinase M2
PLA2G2A: Phospholipase A2 group IIA
PPARα: Peroxisome proliferator-activated receptor α
PPP: Pentose phosphate pathway
Rag: Ras-related GTP-binding
RAS: Rat sarcoma
ROS: Reactive oxygen species
RLR: RIG-I-like receptor
SAM: S-adenosylmethionine
SASP: Senescence-associated secretory phenotype
SIRT1: Sirtuin 1
SOAT1: Sterol O-acyltransferase 1
SOX9: SRY-box transcription factor 9
STAT3: Signal transducer and activator of transcription 3
STING: Stimulator of interferon genes
SURF4: Surfeit locus protein 4
TAGLN2: Transgelin 2
TAMs: Tumor-associated macrophages
TBK1: TANK-binding kinase 1
TCA: Tricarboxylic acid cycle
TFAM: Mitochondrial transcription factor A
TFEB: Transcription factor EB
TLR4: Toll-like receptor 4
TME: Tumor microenvironment
TNF-α: Tumor necrosis factor-alpha
TRAF2: TNF receptor-associated factor 2
TREM2: Triggering receptor expressed on myeloid cells 2
TREX1: Three prime repair exonuclease 1
TRIM24: Tripartite motif containing 24
TRPV2: Transient receptor potential vanilloid 2
Tregs: Regulatory T cells
ULK1: Unc-51-like kinase 1
UPR: Unfolded protein response
VRAC: Volume-regulated anion channel
VEGF-A: Vascular endothelial growth factor A
VHL: Von Hippel–Lindau
YBX1: Y-box binding protein 1
ZAK: Leucine-zipper and sterile-α motif kinase
α-KG: α-Ketoglutarate
γH2AX: Phosphorylated H2A histone family member X
